# Effectiveness and cost effectiveness of pharmacological thromboprophylaxis for medical inpatients: decision analysis modelling study

**DOI:** 10.1136/bmjmed-2022-000408

**Published:** 2024-02-21

**Authors:** Sarah Davis, Steve Goodacre, Daniel Horner, Abdullah Pandor, Mark Holland, Kerstin de Wit, Beverley J Hunt, Xavier Luke Griffin

**Affiliations:** 1 Sheffield Centre for Health and Related Research, School of Medicine and Population Health, University of Sheffield, Sheffield, UK; 2 Department of Emergency and Intensive Care Medicine, Northern Care Alliance Foundation Trust, Salford, UK; 3 Division of Immunology, Infection, Immunity and Respiratory Medicine, University of Manchester, Manchester, UK; 4 School of Clinical and Biomedical Sciences, Faculty of Health and Wellbeing, University of Bolton, Bolton, UK; 5 Department of Emergency Medicine, Queen's University, Kingston, ON, Canada; 6 Department of Thrombosis & Haemostasis, Kings Healthcare Partners, London, UK; 7 Barts Bone and Joint Health, Blizard Institute, Barts and the London School of Medicine and Dentistry, Queen Mary University of London, London, UK

**Keywords:** Thromboembolism, Risk management, Economics, Anticoagulants

## Abstract

**Objective:**

To determine the balance of costs, risks, and benefits for different thromboprophylaxis strategies for medical patients during hospital admission.

**Design:**

Decision analysis modelling study.

**Setting:**

NHS hospitals in England.

**Population:**

Eligible adult medical inpatients, excluding patients in critical care and pregnant women.

**Interventions:**

Pharmacological thromboprophylaxis (low molecular weight heparin) for all medical inpatients, thromboprophylaxis for none, and thromboprophylaxis given to higher risk inpatients according to risk assessment models (Padua, Caprini, IMPROVE, Intermountain, Kucher, Geneva, and Rothberg) previously validated in medical cohorts.

**Main outcome measures:**

Lifetime costs and quality adjusted life years (QALYs). Costs were assessed from the perspective of the NHS and Personal Social Services in England. Other outcomes assessed were incidence and treatment of venous thromboembolism, major bleeds including intracranial haemorrhage, chronic thromboembolic complications, and overall survival.

**Results:**

Offering thromboprophylaxis to all medical inpatients had a high probability (>99%) of being the most cost effective strategy (at a threshold of £20 000 (€23 440; $25 270) per QALY) in the probabilistic sensitivity analysis, when applying performance data from the Padua risk assessment model, which was typical of that observed across several risk assessment models in a medical inpatient cohort. Thromboprophylaxis for all medical inpatients was estimated to result in 0.0552 additional QALYs (95% credible interval 0.0209 to 0.1111) while generating cost savings of £28.44 (−£47 to £105) compared with thromboprophylaxis for none. No other risk assessment model was more cost effective than thromboprophylaxis for all medical inpatients when assessed in deterministic analysis. Risk based thromboprophylaxis was found to have a high (76.6%) probability of being the most cost effective strategy only when assuming a risk assessment model with very high sensitivity is available (sensitivity 99.9% and specificity 23.7% *v* base case sensitivity 49.3% and specificity 73.0%).

**Conclusions:**

Offering pharmacological thromboprophylaxis to all eligible medical inpatients appears to be the most cost effective strategy. To be cost effective, any risk assessment model would need to have a very high sensitivity resulting in widespread thromboprophylaxis in all patients except those at the very lowest risk, who could potentially avoid prophylactic anticoagulation during their hospital stay.

**WHAT IS ALREADY KNOWN ON THIS TOPIC**

- Medical inpatients are at risk of venous thromboembolism, which can be life threatening or result in long term complications; but this condition can be reduced by offering thromboprophylaxis (low molecular weight heparin) to eligible patients (ie, those without contraindications or high bleeding risk)
- It is widely presumed that not all patients benefit from thromboprophylaxis; risk assessment models help clinicians further select medical inpatients who are at increased risk of venous thromboembolism to receive thromboprophylaxis
- Uncertainty exists over which risk assessment models are optimal, and whether using these models is more cost effective than offering thromboprophylaxis to all eligible medical inpatients

**WHAT THIS STUDY ADDS**

- Offering thromboprophylaxis to all eligible medical inpatients is expected to have lower costs and greater health benefits than using risk assessment models to select higher risk groups for tailored prescribing

**HOW THIS STUDY MIGHT AFFECT RESEARCH, PRACTICE, OR POLICY**

- Future research should focus on identifying patients with a low risk of venous thromboembolism who could forego the default option of thromboprophylaxis for all eligible patients
- These results support a shift towards using an opt-out system for thromboprophylaxis based on simple critera, rather than the current opt-in system

## Introduction

Medical inpatients are at increased risk of venous thromboembolism (VTE), such as lower limb deep vein thrombosis (DVT) and pulmonary embolism, during initial hospital admission and for 90 days after discharge.^1^ While most people make a full recovery following VTE, it can complicate hospital recovery and lead to post-thrombotic syndrome or chronic thromboembolic pulmonary hypertension. It can also increase health resource use and occasionally result in death.

Pharmacological thromboprophylaxis can be used to prevent VTE, but is also associated with a potentially increased risk of bleeding,^2^ including fatal bleeds or non-fatal intracranial haemorrhage, which can result in clinically significant morbidity. The widespread use of thromboprophylaxis in medical patients in hospital incurs substantial healthcare costs. Therefore, the overall balance of costs, benefits, and potential harms of thromboprophylaxis should be assessed. This assessment involves estimating the overall clinical effectiveness of thromboprophylaxis in terms of quality adjusted life years (QALYs) gained (thus weighing the benefits of treatment against the risks), and the cost effectiveness of thromboprophylaxis in terms of the additional costs required to gain additional QALYs.

Targeting pharmacological thromboprophylaxis at those patients with the highest risk of VTE could maximise the benefits in terms of avoiding VTE outcomes, while minimising costs and potential harms. Many risk assessment models (RAMs) derived from internally valid study designs have undergone external validation in cohorts of medical inpatients, with the most commonly assessed being the Padua, Geneva, IMPROVE, and Kucher models.^3^ Such models do not perfectly predict those individuals who will go on to have a VTE, so a trade-off between sensitivity and specificity is required to determine the optimal threshold for providing thromboprophylaxis. In addition, clinical time is needed to administer any risk assessment model and inter-rater reliability is variable.^4 5^ The cost effectiveness of using these models to target thromboprophylaxis has not previously been examined for medical inpatients. The aim of this analysis was to assess the overall effectiveness, cost, and cost effectiveness of alternative strategies for pharmacological thromboprophylaxis in medical inpatients. The strategies included thromboprophylaxis for all eligible inpatients, thromboprophylaxis for none, and thromboprophylaxis targeted at higher risk patients using seven RAMs previously validated in medical cohorts.

## Methods

We developed a decision analytical model to simulate the management of a cohort of medical inpatients according to the different thromboprophylaxis strategies and to estimate the short and long term consequences of each strategy. The model estimates the average health and social care costs incurred and the average QALYs accrued across the cohort to estimate the overall cost effectiveness (cost per QALY gained) of each strategy compared with the next most effective strategy. The costs and QALYs are estimated over the patient's whole lifetime, but a discounting rate was applied (3.5% per annum) because benefits and costs occurring early are valued more than those occurring later.^6^

### Model structure

The model has been developed in collaboration with clinical experts who provided guidance on the selection of model outcomes based on clinical importance and assessed the appropriateness of data sources and model assumptions. Existing published models were presented to the clinical experts to inform this discussion.^7–9^ The chosen approach drew mainly on previous work to evaluate thromboprophylaxis during lower limb immobilisation.^9^ A decision tree model (online supplemental figure 1) was used to estimate the number of patients receiving thromboprophylaxis for each strategy and numbers experiencing fatal pulmonary embolism, non-fatal pulmonary embolism, symptomatic DVT, asymptomatic DVT, and major bleeding over a six month time frame. Symptomatic DVTs and non-fatal pulmonary embolisms are assumed to require three months of anticoagulant treatment in accordance with national guidance in England.^10^ The six month time frame was considered sufficient to capture both the period of risk for hospital acquired VTE (90 days after admission) and the period of treatment following VTE (three months), during which time patients are also at risk of major bleeding.

10.1136/bmjmed-2022-000408.supp1 Supplementary data

Major bleeds were divided into fatal bleeds, non-fatal intracranial haemorrhages, and other major bleeds. Patients with major bleeds during either thromboprophylaxis or VTE treatment with anticoagulants are assumed to stop their anticoagulant treatment at the time of the bleed. The likelihood of VTE was assumed to be independent of whether the patient had major bleeding during hospital admission. Major bleeding during hospital stay and with VTE treatment were assumed to be independent events, given the differing doses (between prophylaxis and treatment anticoagulation) and the fact that the model is attempting to estimate average outcomes across the population. Post-thrombotic syndrome and chronic thromboembolic pulmonary hypertension can be difficult to distinguish from acute symptoms during the first three months after VTE, so diagnosis of these chronic complications was assumed not to occur until the end of the decision tree phase of the model. Heparin induced thrombocytopenia was not included in the model because the most important consequence of this condition is an increased risk of VTE. However, any increased VTE risk in patients with heparin induced thrombocytopenia would have contributed to the VTE risk in the prophylaxis arm of the clinical trials and is therefore already accounted for in the model.

A Markov model (online supplemental figure 2) was then used to extrapolate lifetime outcomes including overall survival and ongoing morbidity related to either intracranial haemorrhage or VTE. The Markov model captures the risk of post-thrombotic syndrome following VTE and the risk of chronic thromboembolic pulmonary hypertension following pulmonary embolism. The risk of post-thrombotic syndrome depends on whether the DVT is symptomatic and treated, or is asymptomatic and untreated, and depends also on its location (proximal or distal). Patients experiencing chronic thromboembolic pulmonary hypertension are divided into medical and surgical management to allow for differential costs and survival between these groups. There is also a health state to capture ongoing morbidity following intracranial haemorrhage. Further adverse outcomes (post-thrombotic syndrome, chronic thromboembolic pulmonary hypertension) are not modelled after intracranial haemorrhage, because lifetime costs and QALYs are assumed to be predominantly determined by morbidity related to intracranial haemorrhage. Recurrent VTEs do not appear within the Markov model because these were not expected to differ according to whether patients received thromboprophylaxis during their hospital stay. The Markov model has a six month cycle to extrapolate the outcomes of the decision tree up to one year, followed by annual cycles thereafter. All cause mortality during the first year is applied at six months. Thereafter, the health state occupancy is half-cycle corrected such that all transitions between states, including mortality, are assumed to occur mid-cycle.

### Population

The population was acutely ill medical patients in hospital excluding critical care patients, children (under age 18 years), and pregnant women. The patient groups excluded are known to have VTE risk profiles that differ substantially from the general inpatient population; any risk or effectiveness estimates provided through data evaluating the use of generic RAMs in medical inpatients will not be valid within such populations. Patients identified to be at increased risk of active bleeding, or in whom thromboprophylaxis is contraindicated for other reasons, were excluded from studies used to estimate risks of VTE and bleeding.^11–14^ Such patients are also ineligible for pharmacological thromboprophylaxis in real world populations and were therefore excluded from the model under all strategies. The population characteristics at baseline (age 65.8 years and 44.5% male sex) were based on average characteristics in a cohort of medical inpatients.^15^

### Risk assessment models

**Figure 1:**
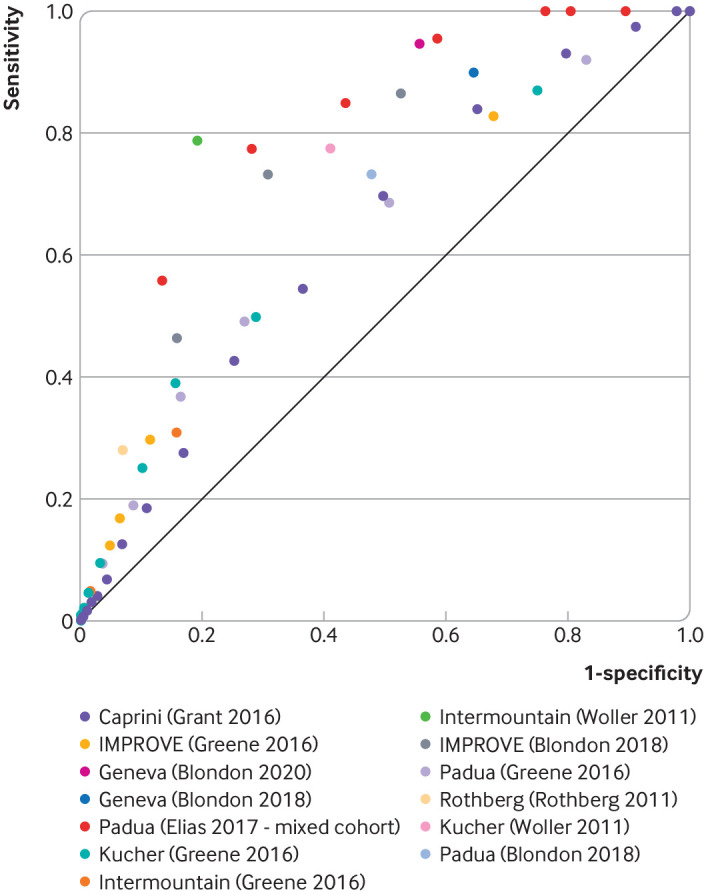
Receiver operator characteristics curve for risk assessment models to predict venous thromboembolism in eligible medical inpatients.^15–20^ Coloured dots refer to model name (and evaluation study). Data from an alternative study^54^ that recruited a mixed cohort of medical and surgical inpatients are also included

The sensitivity and specificity of RAMs for predicting VTE risk, which determines the number receiving thromboprophylaxis, were derived from our previous systematic review of the clinical literature.^3^ This review had identical population inclusion criteria and was intended to directly inform this cost effectiveness work. Available data (summarised in figure 1) suggest that published RAMs generally have weak predictive performance for VTE in medical inpatients, although the studies evaluated were at high risk of bias and were heterogenous in population, design, and ascertainment of VTE cases.^3^ Also, there are clear examples of heterogeneity in estimates of RAM performance when the same RAM is evaluated in different cohorts (eg, Intermountain in Woller 2011, and IMPROVE in Blondon 2018 when compared with their respective performances in Greene 2016).^15–17^ However, more consistency is seen among the performance of five different RAMs (Padua, Caprini, Intermountain, Kucher, IMPROVE) when evaluated in the same cohort,^15 18^ which suggests that any apparent differences in RAM performance are likely to be explained by differences in study design rather than differences between RAMs. Therefore, rather than try to identify the most cost effective RAM, we used regression to explore the trade-off between sensitivity and specificity for a typical RAM. The regression was informed by data from the five RAMs evaluated in a single cohort.^15 18^ Additional details on the regression are provided in the appendix (online supplemental text 2 and figure 3). The performance of the five individual RAMs has been evaluated in the deterministic base case in addition to using the sensitivity and specificity values obtained from the regression. A secondary analysis has also been conducted examining individual estimates of RAM performance for these five RAMs and two additional RAMs (Geneva and Rothberg) externally validated in four other cohorts of medical inpatients.^16 17 19 20^ National UK guidance currently recommends VTE risk assessment for medical inpatients, and the most commonly used tool is the Department of Health's VTE risk assessment tool.^10 21^ However, no data were available on the performance of this tool, so the cost effectiveness of using this specific RAM could not be modelled.^3^

### Thromboprophylaxis and treatment of venous thromboembolism

Pharmacological thromboprophylaxis was assumed to be with subcutaneous, low molecular weight heparin (LMWH) at the dose licensed for medical inpatients for the duration of the hospital admission. Although national guidance has recommended that LMWH is given for a minimum of seven days,^10^ a survey of 25 UK exemplar centres suggests that the majority of hospitals give this treatment for the duration of hospital admission only,^22^ which is typically five days.^23^ It is assumed that the lowest cost preparation is prescribed and that 2.5 minutes of nursing time is required per dose administered. A scenario analysis was conducted to explore the impact of assuming a further two days of LMWH treatment after hospital discharge to achieve a minimum of seven days of thromboprophylaxis. Anticoagulant treatment for subsequent VTEs was assumed to be either phased anticoagulation (LMWH followed by warfarin) or direct oral anticoagulants; a 60:40 split was assumed, based on registry data,^24^ with more widespread use of direct oral anticoagulants explored in a scenario analysis.

LMWH effectiveness was estimated by conducting a random effects meta-analysis of VTE outcomes from three studies giving a relative risk of VTE of 0.49 (95% confidence interval 0.37 to 0.67; online supplemental figure 4).^12–14^ These studies were identified from a published review as being relevant, because they compared standard dose LMWH with placebo in medical inpatients and reported both pulmonary embolism and DVT outcomes, allowing the relative risk of VTE to be estimated.^10^ The relative risk of major bleeding from these three studies was taken directly from a published review (relative risk 1.53, 95% confidence interval 0.8 to 2.92).^10^

### Epidemiological parameters

Data on the absolute risks of DVT, fatal pulmonary embolism, non-fatal pulmonary embolism, fatal bleeding, non-fatal major bleeding (including intracranial haemorrhage), post-thrombotic syndrome, and chronic thromboembolic pulmonary hypertension were obtained from the literature.^8 11–14 25–34^ Patients were assumed to have an increased risk of mortality compared with the general population in the year after hospital admission, in the first six years following intracranial haemorrhage and when experiencing chronic thromboembolic pulmonary hypertension.^35–39^ The clinical parameters incorporated in the model are summarised in table 1, with further details provided in the appendix (online supplemental text 1 and table 1).

**Table 1:** Key clinical parameters incorporated in the decision analytical model to predict clinical outcomes for alternative thromboprophylaxis strategies in eligible medical inpatients*

Parameter description	Value (95% CI)
Absolute risk of VTE in 90 days after hospital admission, without thromboprophylaxis (%)	
Pulmonary embolism	1.38 (0.72 to 2.24)
Symptomatic DVT	2.02 (1.21 to 2.97)
Asymptomatic DVT	30.46 (16.90 to 50.87)
Absolute risk of VTE in 90 days after hospital admission, with thromboprophylaxis (low molecular weight heparin) (%)	
Pulmonary embolism	0.68 (0.34 to 1.18)
Symptomatic DVT	0.99 (0.54 to 1.63)
Asymptomatic DVT	14.93 (7.72 to 27.79)
Major bleed risk by type for medical inpatients without thromboprophylaxis (up to 90 days after admission) (%)	
Fatal major bleeding	0.10 (0.03 to 0.23)
Intracranial haemorrhage	0.06 (0.02 to 0.14)
Other major bleeding	0.51 (0.23 to 1.07)
Any major bleeding	0.67 (0.30 to 1.40)
Major bleed risk by type, for medical inpatients having thromboprophylaxis (up to 90 days after admission) (%)	
Fatal major bleeding	0.15 (0.07 to 0.26)
Intracranial haemorrhage	0.09 (0.03 to 0.16)
Other major bleeding	0.79 (0.50 to 1.14)
Any major bleeding	1.02 (0.65 to 1.47)
Major bleed risk by type, for patients having three months of anticoagulant treatment after VTE (%)	
Fatal major bleeding	0.21 (0.03 to 0.49)
Intracranial haemorrhage	0.08 (0.01 to 0.19)
Other major bleeding	0.56 (0.09 to 1.32)
Any major bleeding	0.85 (0.15 to 1.99)
Case fatality rate for pulmonary embolism (%)	26.8 (11.3 to 33.1)
Standardised mortality ratio *v* general population	
1 year following hospital admission	9.4 (8.9 to 10.0)
2-6 years following intracranial haemorrhage†	2.2 (1.8 to 2.7)
Cumulative 3 year risk of post-thrombotic syndrome for DVT (%)	
Symptomatic proximal location (treated)	32.4 (22.1 to 43.6)
Asymptomatic proximal location (untreated)	56.5 (36.5 to 73.8)
Distal location (symptomatic and treated, or asymptomatic and untreated)	15.6 (7.9 to 25.3)
Cumulative 2 year incidence of chronic thromboembolic pulmonary hypertension (%)	3.2 (2.0 to 4.4)

*Sources described in full in online supplemental table 1.

†Standardised mortality ratio for non-fatal intracranial haemorrhage in year after intracranial haemorrhage was 4.5, so the ratio for medical inpatients was applied in first year after intracranial haemorrhage.

CI, confidence interval; DVT, deep vein thrombosis; VTE, venous thromboembolism.

### Resource use and costs

Costs were assessed from an NHS and Social Services in England perspective and are reported in pound sterling based on 2020 prices. Resource use and unit costs were based on standard NHS sources and published estimates, with historical estimates uplifted using standard healthcare specific inflation indices.^40–45^ We assumed that any patient with VTE during their original medical admission would have their length of stay extended by a duration similar to the duration of admission for patients having VTE after discharge. Use of a RAM by a hospital physician was assumed to take 5 minutes. Costs applied in the model are summarised in table 2 with additional information on resource use in the appendix (online supplemental text 1 and tables 2–4).

**Table 2:** Summary of cost and utility parameters used in the decision analytical model comparing alternative thromboprophylaxis strategies in eligible medical inpatients*

Parameter description	Cost	Utility‡
Application of risk assessment model to patient	£9.08	Not applicable
Thromboprophylaxis†	£23.91	Decrement of 0.007 applied during thromboprophylaxis
Well patient without symptomatic VTE or major bleeding	NA	0.800 in year 1, with age adjustment thereafter
Symptomatic proximal DVT	£763.12	0.769 up to six months; decrement of 0.011 during anticoagulant treatment; beyond six months, multiplier applied only to those individuals with post-thrombotic syndrome
Symptomatic distal DVT	£642.95	
Non-fatal pulmonary embolism	£1848.75	0.768 up to six months; decrement of 0.011 during anticoagulant treatment; beyond 6 months, multiplier applied only to those individuals with chronic thromboembolic pulmonary hypertension
Fatal pulmonary embolism	£1517.13	0
Fatal bleed	£1865.51	0
Non-fatal, non-intracranial bleed	£1209.75	0.685 for one month after bleed
Non-fatal intracranial haemorrhage	£21 987.80 in first 90 days; £8292.83 per year thereafter	0.580 in first six months; multiplier of 0.888 thereafter
Post-thrombotic syndrome	£293.16 in year 1; £78.00 in each subsequent year	Multiplier of 0.895
Medically managed chronic thromboembolic pulmonary hypertension	£18 569.53 each year	Multiplier of 0.629
Surgically managed chronic thromboembolic pulmonary hypertension	£10 236.60 in year 1; £0 in year 2 onwards	Multiplier of 0.629

Costs are based on 2020 prices. £1 (€1.17; $1.26).

*Sources described in full in online supplemental tables 2–6.

†Five days of low molecular weight heparin (dalteparin) for medical inpatients, administered by a hospital nurse (band six).

‡An individual's health utility is a measure of health related quality of life on a scale of 0-1, where 1 represents full health and 0 represents a state equivalent to death.

DVT, deep vein thrombosis; NA, not applicable; VTE, venous thromboembolism.

### Health related quality of life

In order to estimate QALYs, it is necessary to quantify an individual's health utility, which is a measure of health related quality of life on a scale of 0-1, where 1 represents full health and 0 represents a state equivalent to death. General population utility values were applied to those individuals not having any adverse clinical outcomes.^46^ Lifelong utility decrements were applied following intracranial haemorrhage, pulmonary embolism, post-thrombotic syndrome, and chronic thromboembolic pulmonary hypertension. Reductions in utility were applied up to six months for those patients with DVT, for one month after other major bleeds (non-intracranial bleeds), and for the duration of thromboprophylaxis or anticoagulant treatment. Utility data applied in the model are summarised in table 2 with further details in online supplemental tables 5–7.^47–53^

### Probabilistic sensitivity analysis

We assigned probability distributions to reflect the uncertainty around each parameter input and used Monte Carlo simulation to propagate this uncertainty through the model to quantify the decision uncertainty based on 10 000 sets of parameter samples. As RAM performance was similar across the five models when evaluated in a single cohort, we used sensitivity and specificity estimates from one RAM (Padua) in the probabilistic sensitivity analysis. Rather than including the uncertainty in the sensitivity and specificity of this single model within the probabilistic sensitivity analysis, which is likely to under-represent the uncertainty related to RAM performance, the sensitivity and specificity values from the Padua RAM were fixed in the probabilistic sensitivity analysis and the uncertainty related to the sensitivity and specificity of RAMs was explored through scenario analysis. Details of the distributions assumed for each parameter included in the probabilistic sensitivity analysis can be found in online supplemental tables 1 and 7.

### Scenario analyses

We explored the optimal balance between sensitivity and specificity by fitting a linear regression on the logit scale to the receiver operating characteristic curve for all RAMs evaluated in the cohort reported by Greene et al,^15^ to identify the point on the curve that maximised cost effectiveness when valuing a QALY at either £20 000 (€23 440; $25 270) or £30,000; this range represents the threshold for cost effectiveness generally applied in England.^6^

To explore the impact of uncertainty in several key model estimates, we completed multiple specific scenario analyses. Given the heterogeneity in RAM performance (eg, sensitivity and specificity) across the studies (figure 1), we conducted a scenario analysis to explore whether the use of RAMs would be cost effective, if RAM performance was better than the typical performance reported by Greene et al.^15^ For this analysis, we used estimates of RAM performance for the Padua RAM reported by Elias et al, in a study that recruited a mixed cohort of surgical and medical patients.^54^ Post-thrombotic syndrome following asymptomatic distal DVT is also a potentially important outcome with uncertain incidence. We conducted a sensitivity analysis to determine whether the conclusions differed when assuming a zero incidence of post-thrombotic syndrome in patients with asymptomatic distal DVT. In addition, the utility decrement for post-thrombotic syndrome after DVT was not stratified by post-thrombotic syndrome severity, so we conducted a sensitivity analysis to determine whether the conclusions differed when assuming a smaller utility decrement for post-thrombotic syndrome (2% *v* 10%) estimated by combining registry data on the distribution of post-thrombotic syndrome severity with utility estimates stratified by post-thrombotic syndrome severity.^27 55^ Considerable heterogeneity in the case fatality rate for pulmonary embolism was reported in the literature,^13 14 28–30^ so a range of values (13-67%) were explored in sensitivity analyses. Sensitivity analyses were also conducted to explore the impact of assuming a higher or lower average risk for VTE and bleeding.

### Patient and public involvement

The project team included four patient and public involvement members who contributed to the study design and ensured that patient and public values were reflected in the decision analytical modelling. This work included advice about the importance of capturing the utility decrement associated with LMWH injections and the suitability of RAMs. In addition, the modelling methods and results were presented to a broader patient and public involvement group to ensure that the interpretation of the results was comprehensible and relevant to patients and the public.

## Results

Table 3 shows short and long term clinical outcomes per 10 000 patients when using sensitivity and specificity data for the Padua RAM from medical inpatients. Offering thromboprophylaxis to all medical inpatients results in a lower incidence of serious adverse outcomes (fatal pulmonary embolism, fatal bleeds, and non-fatal intracranial haemorrhages) than thromboprophylaxis for none (42 *v* 53 per 10 000). However, thromboprophylaxis for all medical inpatients also results in an increase in other major bleeds (79 *v* 53 per 10 000). The most common adverse outcome for patients in the long term was post-thrombotic syndrome.

**Table 3:** Predicted number of clinical outcomes per 10 000 eligible medical inpatients for each thromboprophylaxis strategy

Patient group offered thromboprophylaxis	Outcomes at 6 months per 10 000 patients							Outcomes at 5 years per 10 000 patients				
	Fatal PE	Fatal bleed	Non-fatal ICH	Other major bleed	Non-fatal PE	Symptomatic DVT	Asymptomatic DVT	PTS	PE survivor with CTEPH	PE survivor without CTEPH	ICH survivor	Death (from any cause)
None	37	10	6	53	101	201	3041	787	2	83	5	1498
Padua ≥7†	36	10	6	54	98	196	2965	767	2	81	5	1497
Padua ≥6†	35	11	6	54	96	191	2893	749	2	79	5	1497
Padua ≥5†	33	11	6	56	91	181	2747	711	2	75	5	1495
Padua ≥4†	30	11	6	59	82	163	2469	639	2	68	5	1493
Padua ≥3†	28	12	7	62	75	150	2277	589	2	62	5	1491
Padua ≥2†	24	13	7	68	65	130	1975	511	1	54	6	1489
Padua ≥1†	20	14	8	75	53	106	1612	417	1	44	7	1487
All	18	15	9	79	49	98	1489	385	1	41	7	1486

*Patients having other major bleeds could also have deep vein thrombosis or non-fatal pulmonary embolism.

†Numbers denote Padua scores at which thromboprophylaxis is offered to patients; sensitivity and specificity data for each Padua score taken from Greene et al.^15^

CTEPH, chronic thromboembolic pulmonary hypertension; DVT, deep vein thrombosis; ICH, intracranial haemorrhage; PE, pulmonary embolism; PTS, post-thrombotic syndrome.

**Figure 2:**
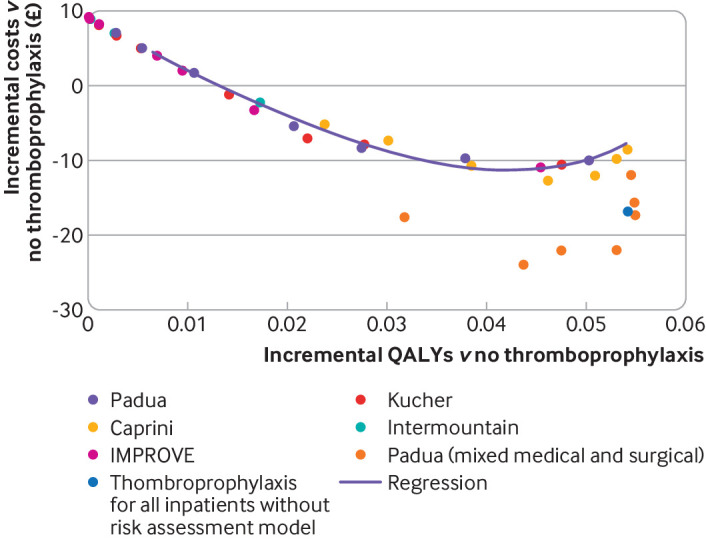
Cost effectiveness plane for offering thromboprophylaxis in eligible medical inpatients according to five risk assessment models. All five models are validated in one cohort of medical inpatients,^15 18^ and the Padua risk assessment model is also validated in another study with mixed cohort of medical and surgical patients.^54^ £1=€1.17; $1.26. Costs are based on 2020 prices. QALY=quality adjusted life years

Figure 2 shows the incremental costs and QALYs, compared with no thromboprophylaxis, that are expected to be achieved for the five RAMs evaluated in the cohort reported by Greene et al.^15^ The multiple points presented for each study reflect the different cut-off scores available, each of which represents a different sensitivity and specificity profile. In addition, the line in figure 2 shows expected costs and QALYs for a typical RAM, based on the linear regression for RAM performance across these five RAMs. In general, a strategy of thromboprophylaxis for all medical inpatients dominates the alternative of using a RAM to determine thromboprophylaxis (ie, has both higher QALYs and lower costs) because QALY gains and cost savings from preventing VTE increase as the proportion of people receiving thromboprophylaxis increases and these gains/savings largely outweigh the additional costs of LMWH. Therefore, the point on the receiver operating characteristic curve that maximises QALY gains and cost savings would have a sensitivity of 100% and a specificity of 0%; this performance is the same as that for thromboprophylaxis for all medical inpatients, but with the added clinical cost of applying a RAM. In the secondary analysis, none of the estimates of model performance in other medical cohorts was sufficient to alter the conclusion that thromboprophylaxis for all medical inpatients is more cost effective than using RAMs (online supplemental figure 5). However, in the scenario analysis exploring higher estimates of model performance, using estimates from Elias et al^54^ offering thromboprophylaxis to only patients with a Padua score of ≥3 was more cost effective than offering it to all patients—the high performance of the RAM in this particular study (99.9% sensitivity) meant that offering thromboprophylaxis to all medical inpatients resulted in additional patients being exposed to bleeding risks with no additional VTEs prevented.

Table 4 presents base case results from the probabilistic sensitivity analysis for the Padua RAM as an example of a typical RAM developed and validated in medical inpatients, although similar results are expected for the alternative models based on figure 2. The regression was not used for the probabilistic sensitivity analysis because it would have been necessary to have arbitrarily selected specific points on the curve to compare. Thromboprophylaxis for all medical inpatients was estimated to result in 0.0552 additional QALYs (95% credible interval 0.0209 to 0.1111) while generating cost savings of £28.44 (−£47 to £105). Thromboprophylaxis for all medical inpatients dominates no thromboprophylaxis in 79.1% of samples from the probabilistic sensitivity analysis, and we estimated a 99.8% probability that the incremental cost effectiveness ratio is under £20 000 per QALY. Table 4 also presents the results of the probabilistic sensitivity analysis for the scenario assuming higher RAM performance based on Elias et al.^54^ In this scenario, offering thromboprophylaxis to only patients with a Padua score of ≥3 has a 76.6% probability of being the most cost effective strategy when valuing a QALY at £20 000, and a corresponding 79.8% probability when valuing a QALY at £30 000.

**Table 4:** Base case results and scenario analysis results for offering thromboprophylaxis in eligible medical inpatients according to the Padua risk assessment model (mean of 10 000 samples from probabilistic sensitivity analysis)

Patient group offered TPX‡	TPX (%)	Sensitivity (%)	Specificity (%)	Costs (£)	No of QALY	Incremental cost effectiveness ratio *v* previous non-dominated* strategy (£)
**Base case results using performance data from a cohort of medical inpatients (Greene 2016)** **15**						
None	0	0	100	244.93	9.0033	Dominated by TPX for all
Padua ≥7	3	5	98	251.40	9.0061	Dominated by TPX for all
Padua ≥6	6	10	96	249.09	9.0087	Dominated by TPX for all
Padua ≥5	12	19	91	244.81	9.0141	Dominated by TPX for all
Padua ≥4	23	37	84	235.91	9.0243	Dominated by TPX for all
Padua ≥3	35	49	73	231.70	9.0311	Dominated by TPX for all
Padua ≥2	57	69	49	227.79	9.0417	Dominated by TPX for all
Padua ≥1	86	92	17	224.43	9.0544	Dominated by TPX for all
All inpatients	100	100	0	216.49	9.0585	Dominates all other strategies
**Scenario analysis using performance data from alternative study (Elias 2017)** **54**†						
None	0	0	100	242.95	9.0031	Dominated by Padua ≥4
Padua ≥7	28	56	87	220.57	9.0351	Dominated by Padua ≥4
Padua ≥6	45	77	72	212.07	9.0471	Dominated by Padua ≥4
Padua ≥5	58	85	57	212.90	9.0510	Dominated by Padua ≥4
Padua ≥4	71	96	41	211.68	9.0567	Dominates TPX for none
Padua ≥3	84	100	24	215.49	9.0586	1918 *v* Padua ≥4
Padua ≥2	87	100	20	217.03	9.0586	Dominated by Padua ≥3
Padua ≥1	93	100	11	220.41	9.0583	Dominated by Padua ≥3
All inpatients	100	100	0	215.31	9.0580	Extendedly dominated

*An intervention is said to dominate another if it has lower costs and higher QALYs.

†Elias et al recruited a mixed cohort of medical and surgical patients rather than an exclusive medical cohort.^54^

‡Numbers denote Padua scores at which thromboprophylaxis is offered to patient

QALYs, quality adjusted life years; TPX, thromboprophylaxis.

In sensitivity analyses (online supplemental table 8), thromboprophylaxis for all medical inpatients continued to dominate thromboprophylaxis for none when applying a lower utility decrement for post-thrombotic syndrome and when applying either a lower or higher case fatality rate for pulmonary embolism. The scenario analysis assuming all VTE events could be treated with direct oral anticoagulants had minimal impact on the results. When assuming no risk of post-thrombotic syndrome from asymptomatic DVT, thromboprophylaxis for all medical inpatients remained the most cost effective strategy (when valuing a QALY at £20 000), but it no longer resulted in lower costs, giving a cost per QALY of £2089 versus no thromboprophylaxis. Similarly, in the scenario analysis assuming that LMWH is administered for seven days, including two days after discharge, thromboprophylaxis for all medical inpatients remained the optimal strategy but had a cost per QALY of £1200 compared with no thromboprophylaxis.

The optimal strategy was relatively robust to changes in the baseline risks of symptomatic VTE and major bleeding, with thromboprophylaxis for all medical inpatients remaining optimal until the risk of VTE was reduced sixfold (from 3.4% to 0.6%) or the risk of bleeding was increased sixfold (from 0.67% to 4.00%). However, thromboprophylaxis for all medical inpatients was no longer a cost saving strategy when the VTE risk halved or the bleeding risk doubled, giving incremental cost effectiveness ratios of £915 per QALY and £426 per QALY, respectively. Two-way sensitivity analysis identified that the optimal strategy was no longer thromboprophylaxis for all medical inpatients when a threefold increase in bleeding risk (2.00%) was combined with a halving of VTE risk (1.7%). If considering only the balance of benefits and harms, thromboprophylaxis for all medical inpatients would result in QALY losses compared with no thromboprophylaxis, in a cohort with a 1.7% risk of symptomatic VTE and a 4% risk of major bleeding without thromboprophylaxis.

## Discussion

### Principal findings

Offering pharmacological thromboprophylaxis to all eligible medical inpatients appears to be more cost effective than using existing RAMs to target thromboprophylaxis at higher risk patients, owing to the weak predictive performance of existing RAMs. However, scenario analysis suggested that using a high sensitivity RAM to select low risk patients who could avoid thromboprophylaxis might be cost effective, if such a RAM could be developed and validated.

A key strength of this de novo economic analysis is the synthesis of evidence on both benefits and harms to explore the trade-off between preventing VTE and the adverse events profile associated with thromboprophylaxis. The results suggest that the benefits of thromboprophylaxis in terms of reducing VTE outweigh the harms of increased bleeding risk in the medical inpatient population. The conclusion that thromboprophylaxis for all medical inpatients is optimal is fairly robust to the changes explored in the scenario and sensitivity analyses. Furthermore, our findings that thromboprophylaxis for all eligible medical inpatients appears dominant renders moot further complex discussions on the appropriate threshold for prescribing.

The inherent value for any clinical decision rule guiding treatment is based entirely on whether it can outperform generic prescribing; given that thromboprophylaxis for all medical inpatients dominated a variety of threshold values with differing sensitivity and specificity characteristics, the discussion on who selects appropriate thresholds for prescribing becomes obsolete. Overall, our findings suggest that it might be better to move towards a default strategy of offering thromboprophylaxis to all eligible medical inpatients. This strategy would be a change from the current system of using RAMs to select higher risk groups for thromboprophylaxis (opt-in).

A cost effectiveness analysis from a US health system perspective found that LMWH is cost effective for medical inpatients when the risk of VTE is over 1%.^7^ Le et al discussed the use of RAMs to identify patients with a risk lower than 1%, but did not explicitly model the cost effectiveness of this strategy by taking into account the performance of specific RAMs.^7^ Furthermore, the US analysis only included patients with pulmonary embolism and symptomatic proximal DVTs in the model; therefore, the results would not be expected to be comparable with our model, which includes both distal DVT and asymptomatic proximal DVT.

### Limitations of the study

A key limitation of our analysis is the heterogeneity in the estimates of RAM performance across the various cohorts. Owing to this heterogeneity, the uncertainty in the performance of RAMs was explored through scenario analysis, rather than incorporating the precision for a single RAM within the probabilistic sensitivity analysis. In the scenario analysis exploring estimates of model performance from an alternative study, the optimal strategy was to use a RAM rather than to offer thromboprophylaxis for all medical inpatients. This change in the optimal strategy was because Elias et al reported a sensitivity of 99.9% and a specificity of 23.7% for a Padua score of ≥3, resulting in 84% of patients receiving thromboprophylaxis.^54^ These findings likely overestimate sensitivity and are in contrast to the sensitivity and specificity values reported by Greene et al for a Padua score of ≥3 in an exclusively medical cohort, which were 49.3% and 73.0%, respectively.^15^ This heterogeneity could reflect differences in the calibration performance of the RAM, whereby patients with a Padua score <3 have a much lower absolute VTE risk in the Elias et al cohort, owing to the mix of medical and surgical patients.

Using a poorly calibrated model might be more harmful than adopting an approach of thromboprophylaxis for all medical inpatients, if it underpredicts VTE risk in patients who are then advised to forgo thromboprophylaxis.^56^ These findings suggest that a RAM would need to be well calibrated and have a high sensitivity to be more cost effective than a strategy of thromboprophylaxis for all and even then, would still likely result in a very high proportion of patients receiving thromboprophylaxis. The Department of Health's model for assessing VTE risk, which has not been validated but has been widely used in the NHS since 2010, results in over 70% of medical inpatients in the UK receiving thromboprophylaxis, with some trusts offering thromboprophylaxis to over 90% of medical inpatients.^10^ This high usage of thromboprophylaxis could be one of the reasons for the reported national improvement in outcome, regarding reduction in post-admission mortality attributable to VTE.^57^

Other limitations include the potential regular use of antiplatelet treatment in a proportion of this cohort and the increasing use of weight adjusted dosing for LMWH agents.^21^ Our key RAM validation studies did not report on single or dual antiplatelet use at baseline, or on weight adjusted dosing of LMWH. As such, we are unable to comment on whether these treatments have any specific incremental impact on VTE and bleeding risk, and our findings should be applied with caution to these groups. In addition, our findings do not evaluate the use of RAMs in patients on any kind of baseline anticoagulant drug treatment and should not be applied to these groups; furthermore, patients receiving anticoagulation treatment are already established on pharmacological thromboprophylaxis, so do not require additional risk assessment. Of note, the costs of one dose of LMWH are essentially identical across weight bands^42^; we therefore do not believe that use of weight adjusted LMWH thromboprophylaxis would substantially affect the results of the model, unless this strategy can be proven to reduce VTE event rates compared with a standard dosing regimen.

One key issue with studies of RAM performance is that the routine use of thromboprophylaxis within observational cohorts could lead to the performance of these models being underestimated, because the VTE events that would have occurred in higher risk patients are prevented by thromboprophylaxis. The estimates of performance from the study by Elias et al were taken from the subset of patients not receiving thromboprophylaxis, which might partly explain the higher estimate of sensitivity and specificity, although Elias and colleagues reported that the performance was similar in the subset of patients receiving thromboprophylaxis.^54^ Given that the available data suggest that widespread use of pharmacological thromboprophylaxis is both beneficial to patients and is cost effective, future studies are likely to involve cohorts with widespread use of thromboprophylaxis, thus making estimation of RAM performance problematic. Future research could focus on randomised studies of pharmacological thromboprophylaxis versus no pharmacological thromboprophylaxis in patients identified as low risk for VTE during hospital admission.

### Conclusion

We found that pharmacological thromboprophylaxis for all eligible medical inpatients is expected to have lower costs and greater health benefits compared with selective thromboprophylaxis based on currently available models assessing the risk of venous thromboembolism. Scenario analyses suggest that for any RAM to be worth using, it would need to achieve a very high sensitivity. Based on these findings, future research should potentially focus on which medical inpatients can safely forego thromboprophylaxis, rather than who should commence it.

## Data Availability

Data are available upon reasonable request. Requests for access to data should be addressed to the corresponding author or to the data custodian (if known).
