# Supplementary material for: Effectiveness and cost effectiveness of pharmacological thromboprophylaxis for medical inpatients: decision analysis modelling study
Source: BMJ Med. 2024 Feb 21;3(1):e000408. doi: 10.1136/bmjmed-2022-000408 (PMC10882286; doi:10.1136/bmjmed-2022-000408)
Supplement: Supplementary data [file bmjmed-2022-000408supp001.pdf]

Decision-analysis modelling of effectiveness and cost-effectiveness of thromboprophylaxis for medical inpatients – Supplementary materials

Appendix

Text 1: Additional details on epidemiological parameters, resource use and utilities

Text 2: Additional details on the regression to estimate typical RAM performance

Appendix Table 1: Clinical parameters (including probabilistic distributions)

Appendix Table 2: Summary of cost parameters

Appendix Table 3: Drug costs for treating DVT and PE

Appendix Table 4: Resource use and costs for patients presenting with PE and symptomatic DVT

Appendix Table 5: Utility values applied in short-term decision tree

Appendix Table 6: Utility multipliers for state-transition phase of the model

Appendix Table 7: Probabilistic distributions for cost and utility inputs

Appendix Table 8: Deterministic one-way sensitivity analyses

Appendix Figure 1: Short-term (six month) decision tree model structure

Appendix Figure 2: Long-term Markov model

Appendix Figure 3: Regression to estimate performance of typical RAM in medical inpatients

Appendix Figure 4: Meta-analysis of VTE outcomes in medical inpatients

Appendix Figure 5: Cost-effectiveness plane for all RAMs validated in cohorts of medical inpatients and the Padua RAM in an alternative study (mixed cohort of medical and surgical inpatients)

Text 1: Additional details on epidemiological parameters, resource use and utilities

The absolute risk of symptomatic VTE in patients not receiving thromboprophylaxis was taken from the risk reported in a prospective observational study by Barbar et al., which was the derivation study for the Padua RAM, using data from the subset not receiving thromboprophylaxis (80% of the cohort). [1] This was considered preferable to using data from the placebo arms of RCTs due to the selective nature of recruitment for RCTs and the age of the studies. The ratio of asymptomatic to symptomatic

DVTs (604:40) and proportion of DVTs that are proximal (31%) were taken from a published model used to inform previous NICE guidance on VTE prevention in hospitalised patients.[2] VTEs are assumed to occur at 42 days post-admission based on data from Barbar et al.[1] The absolute risk of major bleeding during thromboprophylaxis was estimated across the thromboprophylaxis arms of the three RCTs used to estimate the RR of bleeding.[3-5] Bleeds during prophylaxis are assumed to occur half-way through prophylaxis (i.e. 2.5 days). Data from the IMPROVE registry was used to determine the proportions of major bleeds occurring in medical inpatients that are fatal, non-fatal ICH and other major bleeds.[6] The absolute risk of bleeding during anticoagulant treatment, and the proportion of bleeds that are fatal, non-fatal ICH and other major bleeds was based on registry studies in patients having treatment for VTE.[7, 8] The cumulative risk of PTS was also based on a registry study.[9] A study which examined the relationship between PTS and adequate anticoagulation following DVT was used to adjust the risk of PTS in patients with asymptomatic proximal DVT, which is assumed to remain undiagnosed and untreated.[10] The two-year risk of CTEPH in patients surviving three to six months after PE was taken from a systematic review.[11] Based on a prospective study with 10-year follow-up, we assumed that no new case of CTEPH would be diagnosed more than two years after PE.[12] The proportion of patients having medical or surgical management of CTEPH and the long-term survival in each group was taken from a registry study.[13] Patients not having CTEPH, ICH, fatal PEs or fatal bleeds were assumed to have mortality risks equivalent to the general population,[14] except in the first year after hopsital admission where a standardised mortality ratio (SMR) is applied (SMR = 9.4) to reflect the increased mortality risk in the year following a hospital admission.[15] An increased risk of mortality was applied in the first six years following haemorrhagic stroke based on estimates from a retrospective study.[16] The case-fatality rate following PE in medical inpatients (26.8%) was estimated using data from five RCTs,[4, 5, 17-19] identified from a published review, which reported both fatal and non-fatal PE incidence for any thromboprophylaxis or placebo arm.[2]

During the decision tree phase of the model, absolute utility values are applied, with patients who are well having general population utility values and all other patients having values applied according to the adverse consequences experienced (DVT, PE, ICH and non-ICH major bleeds). For PE and DVT, these are applied from the time these are experienced until the end of the decision tree model (i.e. up to six months) whereas non-ICH major bleeds are assumed only to have an adverse impact on utility for one month. In addition, absolute utility decrements are applied during thromboprophylaxis to reflect patients' wishes to avoid daily injections and during anticoagulant treatment to reflect patients' wishes to avoid long-term anticoagulation with warfarin. Patients having ICH were assumed to have reduced HRQoL life-long with separate utility values in the short and long-term models. During the

Markov model phase (i.e beyond six months), patients without long-term sequelae or ongoing symptoms (ICH, PTS, PE with CTEPH, or PE without CTEPH) have general population levels of utility which vary with age, based on UK population norms,[20] and those with sequelae or ongoing symptoms have utility multipliers applied which reduce their utility by a fixed proportion relative to the general population level for their age (e.g. multiplier of 0.888 for ICH reduces age-adjusted utility by 11.2%). DVT without PTS was assumed not to result in any HRQoL reduction beyond six months. Patients having successful surgical treatment of CTEPH were assumed to have the same HRQoL as those with PE without CTEPH after one year.

The previous model on thromboprophylaxis in lower limb immobilisation used utility estimates for PE and DVT from the PREFER-VTE registry study.[21] Updated utility values from the PREFER-VTE registry study were identified in the published literature and these were used to calculate utility multipliers for PE and DVT relative to age /gender matched general population estimates.[22, 23] These were used in preference to the previous values as the updated utility estimates were provided separately for patients with and without cancer allowing the impact of VTE independent of cancer to be estimated. For PE, the utility values compared favourably to general population utility values between six and 12 months, therefore the midpoint utility values applied was 1 with a sampled range of 0.998 to 1.000 applied in the PSA. The assumption applied previously, that utility in the month following a non-fatal non-intracranial major bleed would be similar to utility in the first month after PE, was maintained but the multiplier was updated to use utility in the month after PE from the newly the published estimates from PREFER-VTE. The utility estimates applied for other health states (ICH, CTEPH, PTS) were the same as used in the previous published model for thromboprophylaxis following lower limb injury including the disutility applied for thromboprophylaxis and anticoagulant treatment of VTE.[24-27]

Drug costs were based on the NHS Drug Tariff.[28] In the scenario analysis on giving seven days of thromboprophylaxis, resource use associated with post-discharge administration was based on a published estimate by Menakaya *et al.* [29] This study was also used to estimate the cost of LMWH during phased anticoagulant treatment. Monitoring costs were also included for those receiving either warfarin or DOACs. For DOACs these consisted of one nurse led telephone follow-up at 10 days and one consultant led follow-up at three months to assess need for ongoing treatment. For warfarin, follow-up was assumed to consisted of nine face-to-face visit at non-consultant led anticoagulation service over three months plus a consultant led follow-up at three months to assess need for ongoing treatment.

Davis S, *et al. BMJMED* 2024; 3:e000408. doi: 10.1136/bmjmed-2022-000408

Resource use in patients experiencing a VTE, including GP and Emergency Department (ED) attendance, diagnostics tests and emergency admission, was based on clinical expert opinion using assumptions applied in a previous model for patients having outpatient thromboprophylaxis during lower limb immobilisation.[21] Unit costs for these and for fatal bleeds, non-fatal ICH, non-ICH bleeds, PTS and CTEPH were based on 2018/19 NHS reference costs,[30] or national estimates of unit costs for staff time.[31] Exceptions to this were that the costs of fatal bleeds, non-fatal ICHs and the cost of medical treatment for CTEPH were based directly on published sources.[2, 32] Historical prices used as model inputs were uplifted using the hospital and community health services (HCHS) pay and prices index up to 2016 prices[33] and the NHS cost Inflation Index (NHSCII) thereafter.[31]

Text 2: Additional details on the regression to estimate typical RAM performance

Appendix Figure 3 shows the regression of logit(sensitivity) against logit(1-sensitivity) which was used to estimate the performance of a typical RAM in a cohort of medical inpatients. The data points were taken from five RAMs evaluated in a single cohort of medical inpatients,[34, 35] using a range of thresholds to determine the trade-off between sensitivity and specificity. The model was run deterministically for pairs of sensitivity / specificity estimates taken from multiple points along this curve to generate the estimates of costs and QALYs plotted as the dashed line denoted as ‘Regression’ on Figure 2 and Appendix Figure 5.

Davis S, *et al. BMJMED* 2024; 3:e000408. doi: 10.1136/bmjmed-2022-000408

Appendix Table 1: Clinical parameters (including probabilistic distributions)

Parameter description	Midpoint value	Uncertainty measure	Distribution	Source
Probability of PE in medical inpatients	1.38%	95% CI 0.7% to 2.2%	Beta(13,929)	Barbar 2010[1]
Probability of symptomatic DVT in medical inpatients	2.02%	95% CI 1.2% to 3.0%	Beta(19,923)	Barbar 2010[1]
Proportion of all DVTs that are symptomatic	6.21%	95% CI 4.4% to 8.2%	Beta(40,604)	CG92[2]
Proportion of DVTs that are distal (same proportion applied for symptomatic and asymptomatic DVTs)	69%	95%CI 67% to 71%	=1-Beta(1991,32713)/ Beta(6467,28789)	CG92[2] reports that 31% of all DVTs were proximal as estimated from the RCTs in their review that reported the incidence of both: (1,991/34,704)/(6,467/35,256)=(6%/18%)=31%
Effectiveness of prophylaxis in acutely ill medical inpatients – Risk ratio (RR) for VTE	0.49	95% CI 0.37 to 0.67	Lognormal (-0.40,0.15)	Meta-analysis of VTE events in the three RCTs[3-5] included in NG89[36] for LMWH (standard dose / standard duration) versus placebo in acutely ill medical patients (see Appendix Figure 4)
Risk of major bleeding for PPX in medical inpatients	1.02%	95CI 0.65% to 1.47%	Beta(23,2236)	Incidence of bleeding across the LMWH arms of three RCTs which reported bleeding risk in the systematic review of LMWH versus placebo for acutely ill medical inpatients reported in NG89[36]
Proportion of major bleeding during PPX that is fatal for medical inpatients	14%	95% CI 8% to 23%	Beta(12,71)	Bleeds occurring within 14 days of hospitalisation for medical inpatient (minimum length of stay of three days) from IMPROVE registry – average across cohort regardless of use of PPX or not[6]

Proportion of non-fatal major bleeding during PPX that is ICH for medical inpatients	10%	95% CI 4% to 18%	Beta(7,64)	Bleeds occurring within 14 days of hospitalisation for medical inpatient (minimum length of stay of three days) from IMPROVE registry – average across cohort regardless of use of PPX or not[6]
Relative risk of bleeding for prophylaxis versus none in medical inpatients – HR	1.53	95% CI 0.90 to 2.53	Lognormal (0.43,0.33)	Meta-analysis of VTE events in the three RCTs included in NG89 for LMWH (standard dose / standard duration) versus placebo in acutely ill medical patients[36]
Risk of bleeding during three month anticoagulant treatment for VTE	0.8%	95% CI 0.2% to 2.0%	Beta(3,352)	Six-month incidence pooled across patients with HAS-BLED score of zero or one from Kooiman et al.[7]
Proportion of major bleeds during VTE treatment that are fatal	25%	95% CI 21% to 28%	Beta(135,411)	Based on case-fatality rates for major bleeds within the RIETE registry[8]
Proportion of non-fatal major bleeds during VTE treatment that are ICH	9%	95% CI 6.5% to 11.9%	Beta(37,374)	Based on proportion of major non-fatal bleeds within RIETE registry that were ICH (Nieto *et al.*) [8]
All-cause (non VTE related) mortality for general population not in hospital	Varies by age	Assumed fixed	Not applicable	ONS lifetables[14]<br>Risk applied each year is based on current age and is not adjusted to account for contribution of VTE to population mortality.
SMR for deaths in emergency medical inpatients in year after admission compared with deaths in age and sex matched general population	9.43	Ratio of two sampled death rates<br>11.7 (95%CI 11.6 to 11.8) in general population	Norm(11.7,0.05)	Moore 2018[15]

		108 (95%CI 104.4 to 116.5) in hospitalised medical patients	Norm(108,3.09)	
SMR for patients surviving ICH compared with general population<br>– year one after ICH<br>- years two to six after ICH	 NA<br>- 2.2	 Same as for all hospitalised patients<br>95% CI 1.8 to 2.7	 <br>Log(SMR) = norm(0.8,0.1)	SMR from Fogelholm *et al* (2005)[16] applied for years two to six and then assumed no increased mortality risk<br>Increased risk in year after ICH is assumed to be the same as for all hospital inpatients as the SMR for ICH is lower than for the SMR for all medical inpatients<br>Confidence intervals around SMR not reported so have assumed ±20% on the log scale
Probability of PE being fatal in general medical inpatients	26.8%	95% CI 11.3% to 33.1%	Beta(11,30)	Average case-fatality rate across five RCTs[4, 5, 17-19] reporting both PE and fatal PE incidence in NG89[36]
Cumulative risk of PTS for treated symptomatic DVT at three years<br>- proximal<br>- distal	 - 32.4%<br>- 15.6%	 - 95% CI 22.1% to 43.6%<br>- 95% CI 7.9% to 25.3%	 Beta(23,48)<br>Beta(10,54)	Cumulative incidence at three years based on the TULIPA PLUS registry.[9] Distribution of risk across years one to three based on van Dongen 2005 *et al.*[10] Zero risk assumed from year four onwards
OR for PTS in asymptomatic untreated proximal DVT versus treated proximal DVT	2.71	95% CI 1.44 to 5.1	Log(OR) = norm(0.99, 0.32)	OR from van Dongen *et al.* [10]<br>OR applied to risk for treated asymptomatic DVT to get incidence at three years of 56.6% for proximal

				[this gives a PTS risk of 56.5% (95%CI 29.0% to 79.8%) in asymptomatic untreated proximal DVT]
OR for PTS in asymptomatic distal DVT	1	Fixed	Not applicable	Assumed no increased risk for asymptomatic in distal DVT.
Incidence of CTEPH at two years (converted to annual risk of 1.6%)	3.2%	95% CI 2.0 % to 4.4%	Beta(32,967)	Ende-Verhaar *et al.* [11]based on incidence in those surviving the initial treatment period of three to six months<br><br>Assumed no risk beyond two years based on Pengo *et al.* [12]
Proportion of CTEPH treated surgically	59.5%	95% CI 55.8% to 63.2%	Beta(404,275)	Delcroix *et al.* [13]
Proportion of CTEPH that are surgically treated who also received bridging medical care	30.0%	95% CI 24.6% to 33.5%	Beta(117, 287)	Delcroix *et al.* [13]
Mean hazard for exponential survival curve in medically treated patients with CTEPH	0.1168	SE = 0.0123	Norm(0.1168, 0.0123)	Original data from Delcroix *et al.* but curves taken from Goodacre *et al.* [13, 37]<br><br>(If the death hazard falls below general population values then general population values apply)
Mean and SD for lognormal survival curve in surgically treated patients with CTEPH	Mean = 5.08<br><br>SD = 3.34	SE of mean = 0.574<br><br>SE of SD = 0.399	Multivariate normal	Original data from Delcroix *et al.* but curves taken from Goodacre *et al.* [13, 37]<br><br>(If the death hazard falls below general population values then general population values apply)<br><br>Variance – covariance matrix<br><br>Mean log SD log

				Mean log 0.017708 -0.05572<br>SD log -0.05572 0.230935
Age<br>Age x Age<br>Constant	-0.000172<br>-0.000034<br>0.9584588	SE=0.0003737<br>SE=3.96 x 10^{-6}<br>SE = 0.0077431	Multivariate normal	

Abbreviations: CI, confidence interval; CG, clinical guideline; CTEPH, chronic thromboembolic pulmonary hypertension; CODA, convergence diagnostics and output analysis; DVT, deep vein thrombosis; GI, gastrointestinal; ICH, intracranial haemorrhage; NICE, National Institute for Health and Care Excellence; NMA, network meta-analysis; ONS, Office for National Statistics; OR, odds ratio; PSA, probabilistic sensitivity analysis; PE, pulmonary embolism; PTS, post-thrombotic syndrome; RAM, risk assessment model; RIETE, Computerized Registry of Patients with Venous Thromboembolism;RCT, randomised controlled trial; SD, standard deviation; SE, standard error; SMR, standardised mortality ratio; TULIPA PLUS, Thrombosis and Pulmonary Embolism in Out-Patients – plus; VTE, venous thromboembolism.

Appendix Table 2: Summary of cost parameters

Parameter description	Mean value	95% CI *	Source	Notes
Application of RAM to patient	£9.08	Fixed	Curtis *et al.* [31]	Cost for five minute of hospital consultant time
Prophylaxis for medical<br>– five days of LMWH (Dalteparin) administered by hospital nurse	£23.91	NA	Admin costs from Curtis *et al.* [31]<br>Drug costs based on Drug Tariff [28]	Dalteparin is lowest cost formulation of LMWH based on current Drug Tariff prices. [28]
Treatment of symptomatic proximal DVT	£763.12	£748.04 to £795.10	NHS reference costs[30]<br>Drug Tariff[28]	Clinical expert discussion regarding likelihood resource use, combined with NHS reference cost data for healthcare contacts and drug tariff costs for treatments<br>(see Appendix Table 4 for more detailed costing breakdown).
Treatment of symptomatic distal DVT	£642.95	£621.76 to £668.61	NHS reference costs[30]<br>Drug Tariff [28]	Clinical expert discussion regarding likelihood resource use, combined with NHS reference cost data for healthcare contacts and drug tariff costs for treatments<br>(see Appendix Table 4 for more detailed costing breakdown)
Treatment of non-fatal PE	£1,848.75	£1,816.98 to £1,884.53	NHS reference costs[30]<br>Drug Tariff [28]	Clinical expert discussion regarding likelihood resource use, combined with NHS reference cost data for healthcare contacts and drug tariff costs for treatments

				(see Appendix Table 4 for more detailed costing breakdown)
Fatal PE	£1,517.13	£1,491.37 to £1,542.99	NHS reference costs[30]	As per non-fatal minus drug therapy for PE
Fatal bleed	£1,865.51	£678.86 to £3698.12	Luengo-Fernandez *et al.* [32]	Costs of fatal haemorrhagic stroke from OXVASC subgroup with atrial fibrillation.<br>Uplifted to current prices using inflation indices
Non-fatal non-ICH bleed	£1,209.75	£1199.79 to £1220.07	NHS reference costs [30]	Weighted average of reference costs for gastrointestinal bleed (HRG codes FZ38G – FZ38P)
Post non-fatal ICH - first 90 days	£21,987.80	£17,413.48 to £27,302.45	Luengo-Fernandez *et al.*[32]	Weighted average of costs for non-fatal haemorrhagic strokes<br>Uplifted to current prices using inflation indices
Post non-fatal ICH - post acute (beyond 90 days) costs per annum	£8,292.83	£5,57.42 to £11,613.69	Luengo-Fernandez *et al.* [32]	Average costs across all stroke types (haemorrhagic not reported separately). Includes GP and ED costs and long-term care cost<br>Uplifted to current prices using inflation indices
PTS cost per annum – year one<br>-Mild/moderate	£293.16 in year one	£279.90 to £306.40	NHS reference costs [30]	One first and one follow-up vascular surgery outpatient appointments

-severe				Weighted average of consultant led and non-consultant led outpatient appointments for non-admitted face-to-face first attendance (WF01B) and follow-up (WF01A) for vascular surgery (service code 107)
PTS cost per annum – year two<br>-Mild/moderate<br>-severe	£78.00 in each subsequent year	Fixed	Curtis *et al.* [31]	2 x GP surgery consultations with qualification costs including direct care staff costs at £37 per appointment
CTEPH cost per annum<br>- Medically managed	£18,569.53 each year	Fixed	NICE CG92[2]	Cost in CG92 was £1,219 per four weeks in 2008/09 prices. This was uplifted to 2018/19 prices using inflation indices.<br>Assume treatment lifelong
CTEPH cost per annum<br>- Surgically managed	£10,236.60 in year one and zero in year two onwards	£9,932.52 to £10,557.20	NHS reference costs [30]	Average of DZ02H, DZ02J and DZ02K “Complex thoracic procedures” relating to procedure code L041 “Pulmonary thromboendodartectomy” for elective inpatients including excess bed days<br>In addition, 29% of surgically treated patients require medical bridging therapy for 4.6 months<br>(average cost £1992)

CI, confidence interval; CG, clinical guideline; CTEPH, chronic thromboembolic pulmonary hypertension; DOAC, direct oral anticoagulant; DVT, deep vein thrombosis; ED, emergency department; GI, gastrointestinal; GP, general practitioner; HAS-BLED, Hypertension, Abnormal renal/liver function, Stroke, Bleeding history or predisposition, Labile international normalized ratio, Elderly, Drugs/alcohol concomitantly; HR, hazard ratio; HRG, healthcare resource group; ICH, intracranial haemorrhage; LMWH, low molecular weight heparin; LTRiP(cast), Leiden–Thrombosis Risk Prediction for patients with cast immobilisation score; NHS, national health service; NICE, National Institute for Health and Care Excellence; NMA, network meta-analysis; ONS, Office for National Statistics; OR, odds ratio; OXVASC, Oxford Vascular Study; PE, pulmonary embolism; PTS, post-thrombotic syndrome; RCT, randomised controlled trial; RIETE, The Computerized Registry of Patients with Venous Thromboembolism; SMR, standardised mortality ratio; TULIPA, Thrombosis and Pulmonary Embolism in Out-Patients; SD, standard deviation; SE, standard error; VKA, vitamin K antagonist; VTE, venous thromboembolism;

* except where stated otherwise e.g. SD or SE

Appendix Table 3: Drug costs for treating DVT and PE

**Drug**	**Dosing and delivery**	**Product and cost**[28]	**Drug cost per course**	**Monitoring / administration cost**[30]	**Proportion using treatment**[21]
Apixaban	Initially 10 mg twice daily for seven days, orally. Followed by 5 mg twice daily, orally for the remainder of the three month (91 days) treatment period	Apixaban 5 mg = £53.20 for 56 tablets (cost per tablet is same for 28 tablet pack size)	£186.20	£73 *	20% (half of the 40% using DOACs)
Rivaroxaban	Initially 15 mg twice daily for 21 days, to be taken orally with food. Followed by 20 mg once daily, to be taken orally with food for the remainder of the three month (91 days) treatment period	Rivaroxaban 20 mg = £50.40 for 28 tablets (cost per tablet is same for 15mg and larger and smaller pack sizes)	£201.60	£73 *	20% (half of the 40% using DOACs)
Enoxaparin	1.5 mg/kg every 24 hours by subcutaneous injection until adequate oral anticoagulation established (seven days)<br>i.e. 120 mg if assuming weight of 80kg	Clexane Forte 120mg/0.8ml solution (Sanofi) - £87.93 for 10 pre-filled syringes Prescription only medicine<br>assumed for other drugs	£61.55	£72.71†	30% (45% of heparin use)

Dalteparin	15 000 units (assuming body weight of 80kg) once daily until adequate oral anticoagulation established (seven days)	Dalteparin sodium 15,000 units / 0.6ml solution (Pfizer Ltd / Ennogen Healthcare Ltd / JM McGill Ltd) - £42.34 for five pre-filled syinges	£59.28	£72.71†	18% (35% of heparin use)
Tinzaparin	175 units / kg once daily until adequate oral anticoagulation established (seven days) i.e. 14,000 units if assuming 80kg	Innohep 14,000 units / 0.7ml solution (LEO Pharma) - £83.30 for 10 pre-filled syringes	£58.31	£72.71†	6% (20% of heparin use)
Warfarin	5mg once daily orally for three months (91 days)	Warfarin sodium 5mg (various suppliers) = £0.70 for 28 tablets	£3.22	£238.84‡	60%
**Average across those using DOACs and those using LMWH /VKA**			£115.55	£216.07	**Total: £331.63**

Abbreviations: DVT, deep vein thrombosis; DOAC, direct oral anticoagulant; LMWH, low molecular weight heparin; PE, pulmonary embolism; VKA, vitamin K antagonist

Note: Costing assumes that packs of syringes and packets of tablets can be split between patients by dispensing pharmacy

* Based on one nurse led telephone follow-up (WF01C) at 10 days and one consultant led follow-up (WF01A) at three months to assess need for ongoing treatment[30]

† Based on the costs estimated by Menakaya *et al[29]* with the number of district nurse administrations reduced to reflect shorter duration of treatment (seven days versus six weeks)

‡ based on HRG costs[30] for nine face-to-face visit at non-consultant led anticoagulation service over three months (WF01B for first attendance and WF01A for follow-up) plus a consultant led follow-up at three months to assess need for ongoing treatment

Appendix Table 4: Resource use and costs for patients presenting with PE and symptomatic DVT

	**Proportion using resource**[21]				
	**Non-fatal PE**	**Symptomatic proximal DVT**	**Symptomatic distal DVT**	**Unit cost per patient using this resource**	**Description**
**Healthcare contacts / admission**					
GP visit	20%	50%	50%	£39	GP cost per surgery consultation with qualification costs including direct care staff costs [31]
Ambulance transfer to Emergency Department	60%	10%	0%	£257	NHS Schedule for Reference Costs 2018-2019<br>"See and treat and convey", code ASS02. [30]
Emergency department visit leading to admission	60%	10%	0%	£279	NHS Schedule for Reference Costs 2018-2019<br>VB05Z Type 01 Admitted (Category two investigation with Category three treatment). [30]
Emergency department without admission	40%	90%	100%	£239	NHS Schedule for Reference Costs 2018-2019<br>VB05Z Type 01 Non-admitted (Category two investigation with Category three treatment) [30]
Short stay admission for PE	60%	0%	0%	£1,410	NHS Schedule for Reference Costs 2018-2019<br>Weighted average cost of non-elective inpatient (short and long-stay with excess bed days) for "Pulmonary Embolus with Interventions", codes DZ09J to DZ09N & DZ09P and DZ09Q. [30]

Davis S, *et al. BMJMED* 2024; 3:e000408. doi: 10.1136/bmjmed-2022-000408

Short stay admission for DVT	0%	10%	0%	£904	NHS Schedule for Reference Costs 2018-2019<br>Weighted average cost of non-elective inpatient (short and long-stay with excess bed days) for "Deep Vein Thrombosis" CC score 0 to 12+, codes YQ51A to YQ51E. [30]
Critical care unit stay	10%	0%	0%	£1,028	NHS Schedule for Reference Costs 2018-2019[30]<br>Weighted average cost of adult Critical Care, zero to six or more organs Supported, codes XC01Z to XC01Z. [30]
**Subtotal for healthcare contacts.**	**£1,374**	**£379**	**£259**		
**Diagnostic costs**					
Risk assessment tool (Wells score)	Included in Emergency Department episode so not costed separately				
D-Dimer					
ECG					
Chest x-ray					
Proximal leg vein Ultrasound	0%	100%	100%	£53	NHS Schedule for Reference Costs 2018-2019. RD40Z Outpatient Ultrasound Scan with duration of less than 20 minutes, without contrast £55[30]
CTPA	90%	0%	0%	£108	NHS Schedule for Reference Costs 2018-2019. RD21A Outpatient Computerised Tomography Scan of one area, with post contrast only, 19 years and over[30]

V/Q SPECT	5%	0%	0%	£287	NHS Schedule for Reference Costs 2018-2019. RN08A Outpatient Single Photon Emission Computed Tomography (SPECT), 19 years and over[30]
V/Q planar	5%	0%	0%	£321	NHS Schedule for Reference Costs 2018-2019. RN18A Outpatient Lung Ventilation or Perfusion Scan, 19 years and over[30]
Echocardiogram	20%	0%	0%	£76	NHS Schedule for Reference Costs 2018-2019. RD51A Outpatient simple echocardiogram[30]
**Subtotal for unbundled diagnostics**	£143	£53	£53		
**Subtotal for drug treatment**	£332	£332	£332		See Appendix Table 3 above.
**Total**	£1,849*	£763	£643		

CC, complication or comorbidity; CTPA, computerised tomography pulmonary angiography; DVT, deep vein thrombosis; ECG, electrocardiogram; PE, pulmonary embolism; GP, general practitioner; SPECT, single photon emission tomography; V/Q, ventilation/perfusion

* Fatal PEs are assumed to incur diagnostic and inpatient costs but not VTE treatment costs i.e. total cost of £1,517

Appendix Table 5: Utility values applied in short-term decision tree

Absolute utility value	Absolute utility value	Range	Source	Notes
Well / asymptomatic DVT without prophylaxis	0.800	0.799 to 0.801	Ara and Brazier 2010[20]	Population mean utility values based on average age and sex mix at base-line
Symptomatic proximal or distal DVT	0.769	0.756 to 0.779	Monreal 2019[23]	3.8% reduction relative to well patients based on comparison of average utility over six months for DVT (0.820) versus PE versus utility of matched population norms (0.852)
non-fatal PE	0.768	0.756 to 0.779	Chuang 2019[22]	4.0% reduction relative to well patients based on comparison of average utility over six months (0.804) for PE versus utility of matched population norms (0.838)
non-fatal ICH	0.580	0.540 to 0.619	Luengo-Fernandez 2013[25]	Absolute decrement of 0.22 measured at one month
non-fatal non-ICH bleed	0.685	0.684 to 0.686	Chuang 2019[22]	Assumed same utility decrement for PE and GI bleeds at one month.<br>14% reduction based on utility for PE at one month (0.718) versus utility of matched population norms (0.838) from Chuang 2019[22]
Prophylaxis – absolute decrement applied to utility values of well / asymptomatic DVT	0.007	0.000 to 0.050	Marchetti 2000[26]	Patients willing to trade average of 2.7 days per year to avoid treatment with LMWH
Treatment - absolute decrement applied to utility	0.011	0.000 to 0.083	Marchetti 2000[26]	Patients willing to trade average of four days per year to avoid treatment with warfarin

values for non-fatal PE or symptomatic DVT				
Fatal PE / fatal bleed	0	NA	Assumption	

DVT, deep vein thrombosis; ICH, intracranial haemorrhage; LMWH, low molecular weight heparin; PE, pulmonary embolism

Davis S, *et al. BMJMED* 2024; 3:e000408. doi: 10.1136/bmjmed-2022-000408

Appendix Table 6 Utility multipliers for state-transition phase of the model

Health state (s)	Utility multiplier relative to well	Range	Source	Notes
PE survivor without CTEPH and PE survivor more than one year after surgery for CTEPH	1.000	0.998 to 1.000	Chuang 2019[22]	Average over six to 12 months following PE compared to matched general population norms[22]
Any DVT without PTS	1	NA	Assumption	Supported by Lubberts *et al*[38] systematic review finding no significant HRQoL decrement in nine long-term studies based on SF-36 outcomes
non-fatal ICH	0.888	0.837 to 0.937	Luengo-Fernandez 2013[25]	Multiplier calculated based on absolute decrement of 0.09 at five years (utility values stable from six months to five years) relative to absolute utility for well state
PTS	0.895	0.816 to 0.952	Enden 2013 [24]	Multiplier calculated based on absolute decrement of 0.09 relative to absolute utility for well state of 0.86
CTEPH –first year for surgically managed and every year for medically managed	0.629	0.579 to 0.690	Meads 2008[27]	Multiplier calculated based on comparison of utility for CTEPH (0.56) versus utility for NYHA class I (0.89)

CTEPH, chronic thromboembolic pulmonary hypertension; DVT, deep vein thrombosis; HRQoL, Health-related quality of life ;ICH, intracranial haemorrhage; LMWH, low molecular weight heparin; NYHA, New York Heart Association; PE, pulmonary embolism; PTS, post-thrombotic syndrome

Appendix Table 7: Probabilistic distributions for cost and utility inputs

Parameter description	Midpoint value	Uncertainty measure	Distribution	Source
Ambulance transfer to ED	£257	SE = £11	Gamma(551,0.47)	NHS Schedule for Reference Costs 2018-2019.<br>HRG code, ASS02 See and treat and convey[30]
ED visit leading to admission	£279	SE = £6	Gamma(2210, 0.15)	NHS Schedule for Reference Costs 2018-2019.<br>HRG code: Type 01, leading to admission, VB05Z Emergency Medicine, Category two Investigation with Category three Treatment[30]
ED visit not leading to admission	£239	SE=£4	Gamma(3204, 0.07)	NHS Schedule for Reference Costs 2018-2019.<br>HRG code: Type 01, not leading to admission, VB05Z Emergency Medicine, Category two Investigation with Category three Treatment[30]
DVT admission - weighted average of following HRG costs;				NHS Schedule for Reference Costs 2018-2019.<br>Non-elective inpatient (NEI) and non-elective short stay (NESS) costs for HRG codes covering Deep vein thrombosis with CC scores ranging from 0 to 12+[30]
YQ51A – NEI (N=1,377)	£4,017	SE=£198	Gamma(412, 9.7)	
YQ51A – NESS (N=492)	£564	SE=£33	Gamma(288, 2.0)	
YQ51B – NEI (N=1,183)	£2,873	SE=£129	Gamma(495, 5.8)	
YQ51B – NESS (N=895)	£470	SE=£13	Gamma(1237,0.4)	
YQ51C – NEI (N=1,665)	£2,433	SE=£78	Gamma(973, 2.5)	
YQ51C – NESS (N=2,391)	£418	SE=£11	Gamma(1433,0.3)	
YQ51D – NEI (N=1,686)	£2,020	SE=£46	Gamma(1903,1.1)	

YQ51D – NESS (N=6,249)<br>YQ51E – NEI (N=908)<br>YQ51E- NESS (N=11,731)	£384<br>£1,772<br>£320	SE=£9<br>SE=£42<br>SE=9	Gamma(1822,0.2)<br>Gamma(1814,1.0)<br>Gamma(1330,0.2)	
PE admission- weighted average of following HRG costs;<br>DZ09J – NEI (N=888)<br>DZ09J – NESS (N=62)<br>DZ09K – NEI (N=585)<br>DZ09K – NESS (N=65)<br>DZ09L – NEI (N=3,160)<br>DZ09L – NESS (N=1,181)<br>DZ09M – NEI (N=3,716)<br>DZ09M – NESS (N=2,197)<br>DZ09N – NEI (N=5,105)<br>DZ09N – NESS (N=4,374)<br>DZ09P – NEI (N=6,126)<br>DZ09P – NESS (N=8,768)<br>DZ09Q – NEI (N=3,226)<br>DZ09Q – NESS (N=9,048)	 £5,450<br>£1,280<br>£3,384<br>£790<br>£3,522<br>£667<br>£2,671<br>£577<br>£2,201<br>£533<br>£1,845<br>£488<br>£1,584<br>£448	 SE=£277<br>SE=£168<br>SE=£130<br>SE=£56<br>SE=£140<br>SE=£21<br>SE=£75<br>SE=18<br>SE=£45<br>SE=12<br>SE=£38<br>SE=£12<br>SE=£29<br>SE=9	 Gamma(338,14)<br>Gamma(58, 22)<br>Gamma(676, 5.0)<br>Gamma(199, 4.0)<br>Gamma(663, 5.5)<br>Gamma(1026, 0.7)<br>Gamma(1255,2.1)<br>Gamma(1054,0.6)<br>Gamma(2358,0.9)<br>Gamma(2091, 0.3)<br>Gamma(2417,0.8)<br>Gamma(1595, 0.3)<br>Gamma(2989, 0.5)<br>Gamma(2376, 0.2)	NHS Schedule for Reference Costs 2018-2019.<br>Non-elective inpatient (NEI) costs and non-elective short stay (NESS) costs for HRG codes covering Pulmonary embolus with and without interventions with CC score from 0 to 12+ [30]
Critical care – weighted average of HRG costs for codes;				NHS Schedule for Reference Costs 2018-2019.

23

XC01Z	£1,673	N=1	Fixed	HRG codes for Adult Critical Care for zero to six organs supported[30]
XC02Z	£1,574	SE=£152	Gamma(107, 14.7)	
XC03Z	£1,655	SE=£114	Gamma(211, 7.9)	
XC04Z	£1,640	SE=£67	Gamma(605, 2.7)	
XC05Z	£1,450	SE=£49	Gamma(884, 1.7)	
XC06Z	£792	SE=£78	Gamma(104, 7.6)	
XC07Z	£516	SE=£129	Gamma(16.0, 32.2)	
Proximal leg vein ultrasound	£53	SE=£1	Gamma(2135,0.03)	NHS Schedule for Reference Costs 2018-2019[30]
CTPA	£108	SE=£4	Gamma(635,0.17)	NHS Schedule for Reference Costs 2018-2019<br>RD21A Outpatient Computerised Tomography Scan of one area, with post contrast only, 19 years and over[30]
V/Q SPECT	£287	SE=£20	Gamma(202,1.42)	NHS Schedule for Reference Costs 2018-2019<br>RN08A, Outpatient Single Photon Emission Computed Tomography (SPECT), 19 years and over[30]
V/Q planar	£321	SE=£10	Gamma(1045,0.31)	NHS Schedule for Reference Costs 2018-2019<br>RN18A Outpatient Lung Ventilation or Perfusion Scan, 19 years and over[30]
Echocardiogram	£76	SE=£6	Gamma(146,0.52)	NHS Schedule for Reference Costs 2018-2019

				RD51A Outpatient Simple Echocardiogram, 19 years and over[30]
Proportion receiving LMWH who need district nurse administration	4%	95% CI 1.3% to 7.8%	Beta(5,123)	Menakaya *et al [29]*
Fatal bleed	£1,592	SD=1886, N=8	Gamma(5.70, 279)	Luengo-Fernandez *et al* (cost before inflation)[32]
Acute costs for non-fatal ICH (first 90 days) - Weighted average of;				Luengo-Fernandez *et al* [32] (cost before inflation)
Non-disabling non-fatal stroke	£9,903	SD = 4510, N=5	Gamma(24, 411)	
Moderately-disabling non-fatal stroke	£25,442	SD = 9635, N=3	Gamma(21, 1216)	
Totally-disabling non-fatal stroke	£43,036	SD = NA, N=1	Fixed	
Residential costs for non-fatal ICH (first 90 days)	£6,880	SD=£15,600, N=136	Gamma(26,260)	Luengo-Fernandez *et al [32]*
GP costs for non-fatal ICH (first 90 days)	£98	95% CI £27 to £169	Norm(98,36)	Luengo-Fernandez *et al [32]*
Emergency care costs for non-fatal ICH (first 90 days)	£99	95% CI £56 to £141	Norm (99, 22)	Luengo-Fernandez *et al [32]* (cost before inflation (cost before inflation)
Non-fatal non-ICH bleed (weighted average of HRG costs);				NHS Schedule for Reference Costs 2018-2019

FD03A – NEI (N=1,110)	£5,377	SE=£201	Gamma(714, 7.5)	HRG codes for GI bleed without interventions, with single interventions and with multiple interventions. [30]
FD03A – NESS (N=30)	£2,360	SE=£310	Gamma(58, 41)	
FD03B– NEI (N=885)	£3,510	SE=£131	Gamma(722, 4.9)	
FD03B– NSS (N=16)	£2,088	SE=£1,109	Gamma(3.6, 590)	
FD03C – NEI (N=1,642)	£3,866	SE=£171	Gamma(514, 7.5)	
FD03C– NSS (N=41)	£1,345	SE=£105	Gamma(166, 8.1)	
FD03D – NEI (N=2,329)	£2,796	SE=£92	Gamma(913, 3.0)	
FD03D– NSS (N=46)	££2,360	SE=£156	Gamma(229, 10)	
FD03E – NEI (N=5,481)	£2,247	SE=£47	Gamma(2331, 1.0)	
FD03E – NEI (N=108)	£1,089	SE=£82	Gamma(£178, 6.1)	
FD03F – NEI (N=2,891)	£2,818	SE=£100	Gamma(792, 3.6)	
FD03F – NEI (N=2,213)	£591	SE=£19	Gamma(1000, 0.6)	
FD03G – NEI (N=7,278)	£2,198	SE=£41	Gamma(2931, 0.8)	
FD03G – NEI (N=8,830)	£541	SE=£15	Gamma(1221,0.4)	
FD03H – NEI (N=16,290)	£1,575	SE=£27	Gamma(3523, 0.8)	
FD03H – NEI (N=40,167)	£438	SE=11	Gamma(1640, 0.3)	
Anticoagulant service face to face follow-up consultant led	£53	SE=£5	Norm(53,5.3) with minimum of zero	NHS Schedule for Reference Costs 2018-2019<br>Service code 324 - WF01A non-admitted[30]
Anticoagulant service face to face follow-up non-consultant led	£20	SE=£2	Norm(20,2.0) with minimum of zero	NHS Schedule for Reference Costs 2018-2019<br>Service code 324- WF01A non-admitted[30]

Anticogulant service first face to face attendance non-consultant led	£26	SE=£3	Norm(26,2.6) with minimum of zero	NHS Schedule for Reference Costs 2018-2019<br>Service code 324- WF01B non-admitted[30]
Anticoagulant service non face to face follow-up non-consultant led	£20	SE=£20	Norm(20,2.0) with minimum of zero	NHS Schedule for Reference Costs 2018-2019<br>Service code 324– WF01C non-admitted [30]
Vascular surgery first appointment face to face consultant led	£165	SE=£6	Gamma(759,0.22)	NHS Schedule for Reference Costs 2018-2019<br>Service code 107 – WF01B non-admitted[30]
Vascular surgery follow-up appointment face to face, consultant led	£134	SE=£4	Gamma(942, 0.14)	NHS Schedule for Reference Costs 2018-2019<br>Service code 107 – WF01A non-admitted[30]
Vascular surgery first appointment face to face non consultant led	£132	SE=£11	Gamma(132,1.0)	NHS Schedule for Reference Costs 2018-2019<br>Service code 107 – WF01B non-admitted[30]
Vascular surgery follow-up appointment face to face, non consultant led	£121	SE=£14	Gamma(79, 1.53)	NHS Schedule for Reference Costs 2018-2019<br>Service code 107 – WF01A non-admitted[30]
Surgical management of CTEPH – average of following HRG costs;<br>DZ02H<br>DZ02J<br>DZ02K	 <br>£9,782<br>£7,500<br>£6,506	 <br>SE=£363<br>SE=£300<br>SE=£270	 <br>Gamma(723, 13.5)<br>Gamma(627, 12.0)<br>Gamma(579,11.2)	NHS Schedule for Reference Costs 2018-2019<br>HRG codes for Complex Thoracic Procedures, 19 years and over, with CC Score ranging from 0 to 6+[30]
Disutility for stroke up to six months	-0.22	95% CI -0.26 to -0.18	Norm(-0.22, 0.02)	Luengo-Fernandez *et al* (2013)[25]

Disutility for stroke from six months	-0.09	95% CI -0.13 to -0.05	Norm(-0.09, 0.02)	Luengo-Fernandez *et al* (2013)[25]
Utility immediately after DVT	0.72	SE=0.006	Beta(3977, 1565)	Monreal 2019[23]
Utility immediately after PE	0.72	SE=0.007	Beta(2741, 1080)	Chuang 2019[22]<br>[assumed same SD as observed for patients having DVT in Monreal 2019]
Utility for DVT without PTS	0.86	95% CI 0.823 to 0.903	Beta(248,40.3)	Enden *et al (*2013) [24]
Disutility for PTS versus no PTS after DVT	0.09	95% CI 0.03 to 0.15	Beta(7.78, 78.6)	Enden *et al (*2013) [24]
Utility for CTEPH	0.56	SD=0.29, N=308	Beta(505, 397)	Meads *et al* (2008)[27]
Utility for NYHA class 1	0.86	SD=0.17, N=35	Beta(105, 12.9)	Meads *et al* (2008)[27]
Utility for LMWH	0.993	SD=0.016	Beta(27.5, 0.205)	Marchetti *et al* (2001) [26]
Utility for warfarin	0.989	SD=0.024	Beta(17.6, 0.195)	Marchetti *et al* (2001)[26]
Utility regression for age related decrement – coefficients for<br>Age<br>Age x Age<br>constant	 -0.000172<br>-0.000034<br>0.9584588	 SE=0.0003737<br>SE=3.96×10^{-6}<br>SE = 0.0077431	Multivariate normal	Ara and Brazier (2011)[20]<br><br>Variance – covariance matrix<br>(columns: Age, Age x Age, constant)<br>Age: 1.4×10^{-7}<br>Age x Age: -1.5×10^{-9}, 1.6×10^{-11}<br>constant: -2.80×10^{-6} 2.8×10^{-8} 6×10^{-5}

Abbreviations: CC, complications and comorbidities; CI, confidence interval; CG, clinical guideline; CTEPH, chronic thromboembolic pulmonary hypertension; CODA, convergence diagnostics and output analysis; CTPA, computerised tomography pulmonary angiography; DVT, deep vein thrombosis; ED, emergency department; GI, gastrointestinal; GP, general practitioner; HR, hazard ratio; HRG, healthcare resource group; ICH, intracranial haemorrhage; IQR, interquartile range; LMWH, low molecular weight heparin; NA, not applicable; NHS, National Health Service; NICE, National Institute for Health and Care Excellence; NMA, network meta-analysis; ONS, Office for National Statistics; OR, odds ratio; PE, pulmonary embolism; PTS, post-thrombotic syndrome; RCT, randomised controlled trial; SD, standard deviation; SE, standard error; SPECT, Single Photon Emission Computed Tomography; V/Q, ventilation – perfusion VTE, venous thromboembolism

Appendix Table 8: Deterministic one-way sensitivity analyses (assumes RAM is Padua and performance is based on Greene et al.)

Scenario	Parameter varied	Parameter value in basecase scenario	Parameter value in current scenario	Optimal strategy in current scenario*
Basecase	N/A	N/A	N/A	PPX for all
Younger patients	Age	65.8	20	PPX for all
Older patients	Age	65.8	80	PPX for all
Double utility decrement for PPX	Utility decrement	0.007	0.015	PPX for all
Low discounting rate for costs and QALYs	Discount rate	3.5%	1.5%	PPX for all
High discounting rate for costs and QALYs	Discount rate	3.5%	6.0%	PPX for all
Utility decrement of PTS is halved	Utility decrement	10%	5%	PPX for all
Utility decrement of PTS from Lenert et al.	Utility decrement	10%	2%	PPX for all
PTS cost from Caprini	Year 1 cost<br>Year 2 cost	£293<br>£78	£1022<br>£423	PPX for all
Lower case-fatality rate of PE	Case-fatality rate of PE	26.8%	13%	PPX for all
Higher case-fatality rate of PE	Case-fatality rate of PE	26.8%	67%	PPX for all
Increased use of DOACs to treat VTE	% of VTE treatment that is DOACs	40%	100%	PPX for all
No asymptomatic DVTs	Asymptomatic DVT risk	30.5%	0%	PPX for all
No PTS risk in asymptomatic DVT (proximal or distal)	Risk of PTS in;<br>- Proximal<br>- Distal	 57.5%<br>15.6%	 0%<br>0%	PPX for all
Low risk of VTE (one sixth of baseline risk)	Risk of symptomatic VTE	3.4%	0.6%	Padua ≥2

Higher bleed risk during PPX (six times higher)	Risk of bleeding without PPX	0.67%	4.00%	Padua ≥1
Low risk of VTE (one half of baseline risk) combined with higher risk of bleeding (three times higher than baseline)	Risk of symptomatic VTE<br>Risk of bleeding without PPX	3.4%<br>0.67%	1.7%<br>2.00%	Padua ≥1
Low risk of VTE (one half of baseline risk) combined with higher risk of bleeding (six times higher than baseline)	Risk of symptomatic VTE<br>Risk of bleeding without PPX	3.4%<br>0.67%	1.7%<br>4.00%	Padua ≥4 †
Bleed risk during VTE treatment reflects any HAS-BLED score	Risk of major bleeding during anticoagulation	0.9%	2.0%	PPX for all
7 days of LMWH (including 2 days post-discharge)	Cost of PPX	£23.91	£101.03	PPX for all
Higher risk of death in year of admission	Risk of death in year 1	10%	22%	PPX for all
No additional cost for administering RAM	Cost of RAM administration	£9.08	£0	PPX for all
Quadruple cost of administering RAM	Cost of RAM administration	£9.08	£36.33	PPX for all
No increase in inpatient care due to VTE	Distal DVT cost<br>Proximal DVT cost<br>PE cost	£644.88<br>£765.05<br>£1850.68	£386.10<br>£386.10<br>£476.57	PPX for all
VTE occurs 3 days after admission	Time of VTE	42	3	PPX for all
VTE occurs 90 days after admission	Time of VTE	42	90	PPX for all

* Optimal strategy when valuing a QALY at £20,000; † PPX for all has lower QALYs than PPX for none in this scenario

Appendix Figure 1: Short-term (six month) decision tree model structure

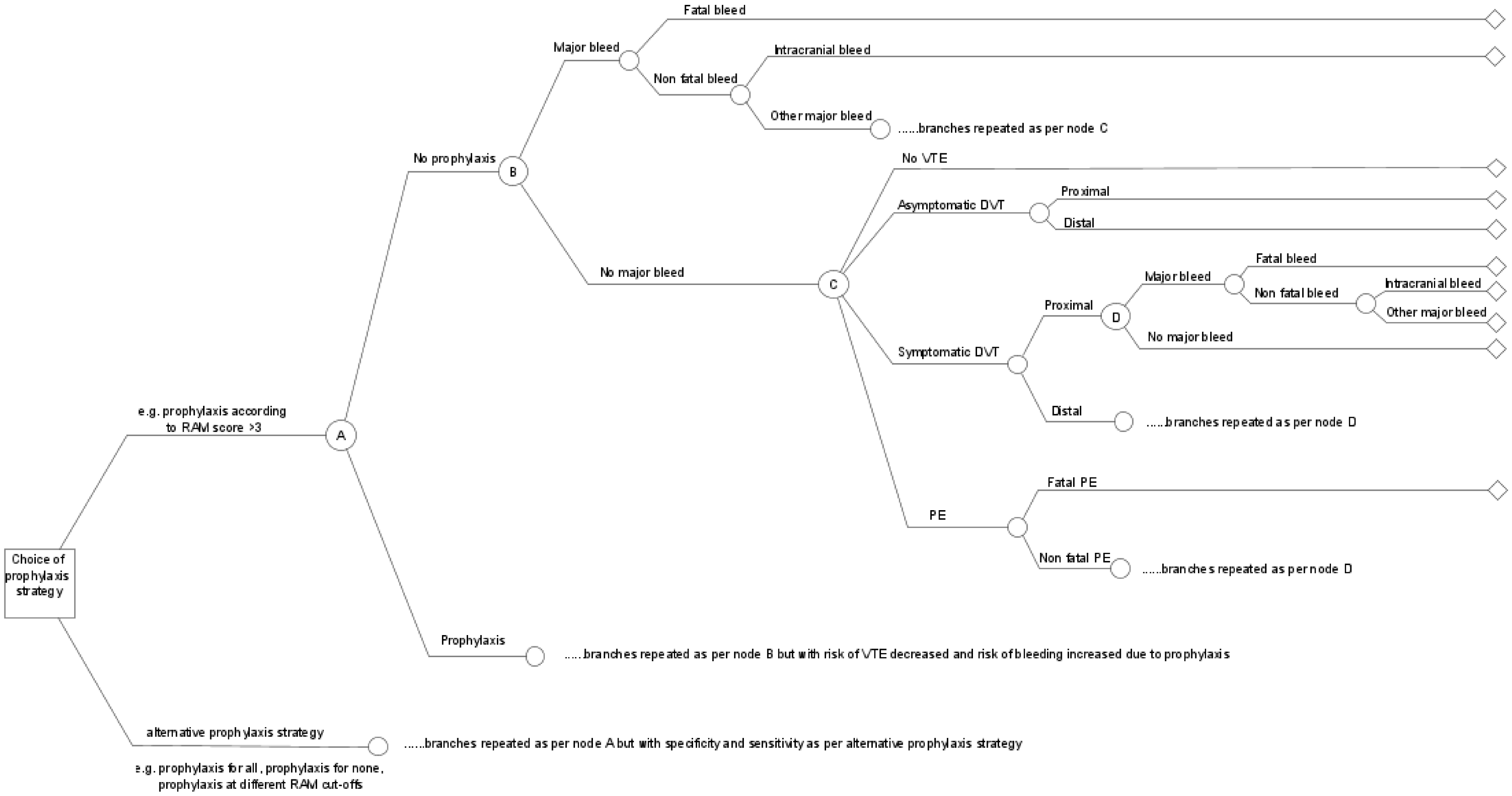

Footnote: Node B captures the risk of bleeding related to prophylaxis whilst Node D represents the risk of bleeding in patients during therapeutic dose anticoagulation given following a symptomatic VTE.

31

Appendix Figure 2: Long-term Markov model (reproduced from Pandor et al.[21])

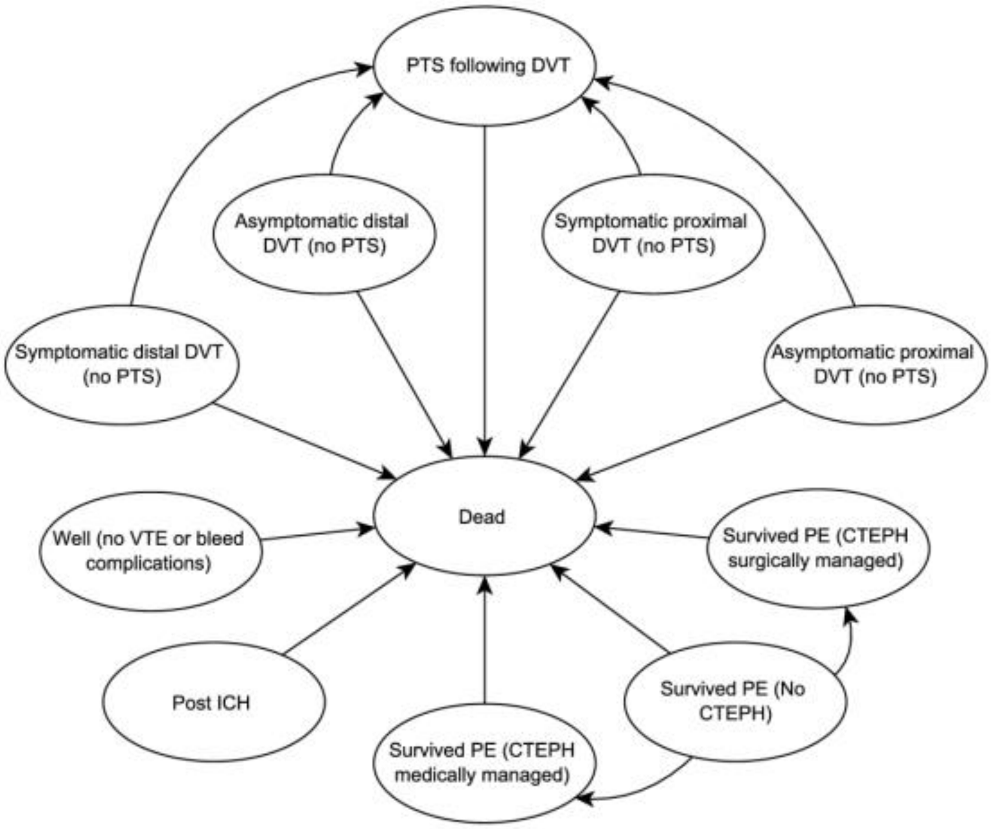

Abbreviations: CTEPH, chronic thromboembolic pulmonary hypertension; DVT, deep vein thrombosis; ICH, intracranial haemorrhage; PE, pulmonary embolism; PTS, post-thrombotic syndrome; VTE, venous thromboembolism

Figure 2 is reproduced from Pandor A, Horner D, Davis S, Goodacre S, Stevens JW, Clowes M, et al. Different strategies for pharmacological thromboprophylaxis for lower-limb immobilisation after injury: systematic review and economic evaluation. Health Technol Assess 2019;23(63)

The copyright statement for the source publication states, "This issue may be freely reproduced for the purposes of private research and study and extracts (or indeed, the full report) may be included in professional journals provided that suitable acknowledgement is made and the reproduction is not associated with any form of advertising. "

Appendix Figure 3: Regression to estimate performance of a typical RAM in medical inpatients

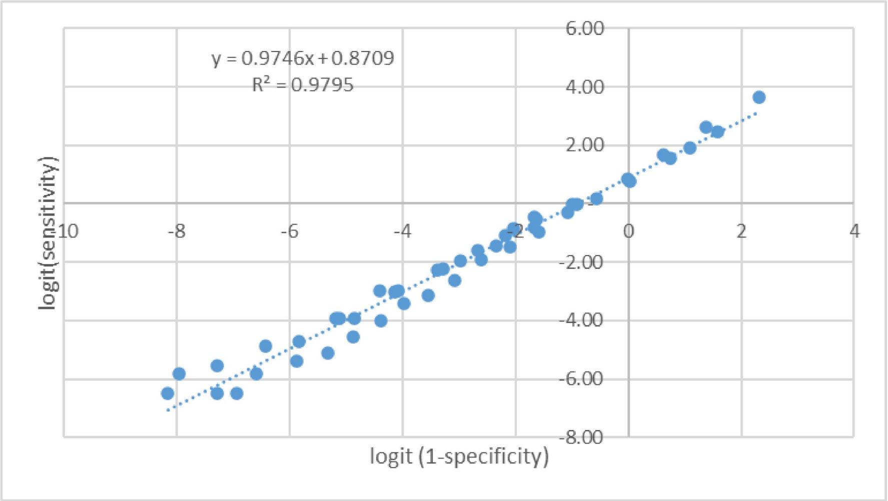

Appendix Figure 4: Meta-analysis of VTE outcomes in medical inpatients

Study or Subgroup	Prophylaxis treatment Events	Prophylaxis treatment Total	Control Events	Control Total	Weight	Risk Ratio M-H, Random, 95% CI
Lederle 2006	5	140	9	140	7.7%	0.56 [0.19, 1.62]
Leizorovicz 2004	42	1518	73	1473	63.3%	0.56 [0.38, 0.81]
Samama 1999	16	291	43	288	29.0%	0.37 [0.21, 0.64]
**Total (95% CI)**		**1949**		**1901**	**100.0%**	**0.49 [0.37, 0.67]**
Total events	63		125			

Heterogeneity: Tau² = 0.00; Chi² = 1.56, df = 2 (P = 0.46); I² = 0%
Test for overall effect: Z = 4.65 (P < 0.00001)

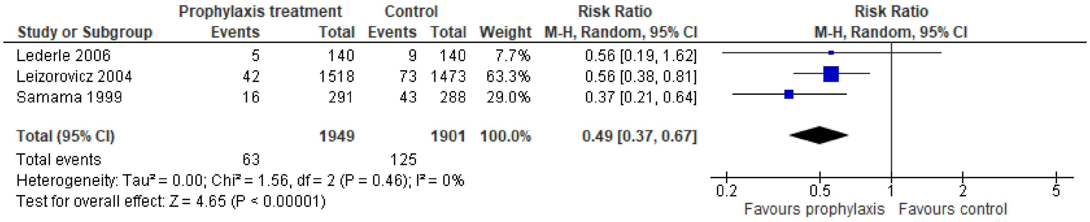

Appendix Figure 5: Cost-effectiveness plane for all RAMs validated in cohorts of medical inpatients and the Padua RAM in an alternative study (mixed cohort of medical and surgical inpatients)

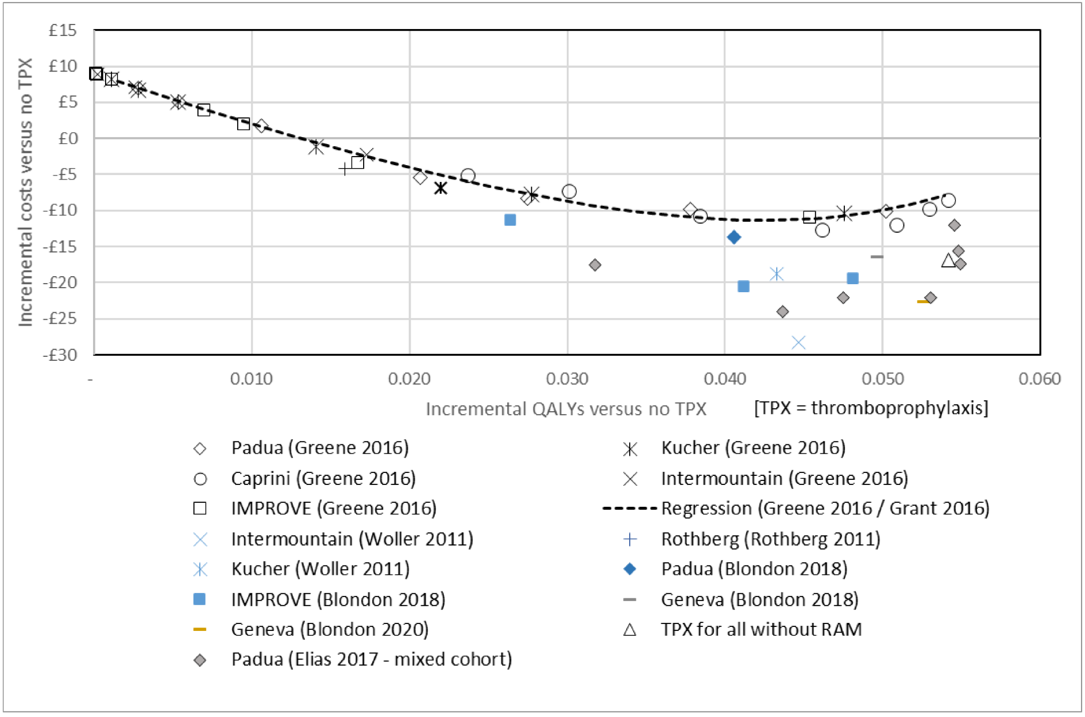

References for supplementary materials

1. Barbar S, Noventa F, Rossetto V, et al. A risk assessment model for the identification of hospitalized medical patients at risk for venous thromboembolism: the Padua Prediction Score. J Thromb Haemost 2010;8(11):2450-7. doi: 10.1111/j.1538-7836.2010.04044.x [published Online First: 2010/08/27]

2. National Clinical Guideline Centre – Acute and Chronic Conditions (UK). Venous Thromboembolism: Reducing the Risk of Venous Thromboembolism (Deep Vein Thrombosis and Pulmonary Embolism) in Patients Admitted to Hospital (NICE Clinical Guidelines, No. 92.): London: Royal College of Physicians (UK), 2010.

3. Lederle FA, Sacks JM, Fiore L, et al. The prophylaxis of medical patients for thromboembolism pilot study. Am J Med 2006;119(1):54-9. doi: 10.1016/j.amjmed.2005.03.049 [published Online First: 2006/01/25]

4. Leizorovicz A, Cohen AT, Turpie AG, et al. Randomized, placebo-controlled trial of dalteparin for the prevention of venous thromboembolism in acutely ill medical patients. Circulation 2004;110(7):874-9. doi: 10.1161/01.CIR.0000138928.83266.24 [published Online First: 2004/08/04]

5. Samama MM, Cohen AT, Darmon JY, et al. A comparison of enoxaparin with placebo for the prevention of venous thromboembolism in acutely ill medical patients. Prophylaxis in Medical Patients with Enoxaparin Study Group. N Engl J Med 1999;341(11):793-800. doi: 10.1056/NEJM199909093411103 [published Online First: 1999/09/09]

6. Decousus H, Tapson VF, Bergmann JF, et al. Factors at admission associated with bleeding risk in medical patients: findings from the IMPROVE investigators. Chest 2011;139(1):69-79. doi: 10.1378/chest.09-3081 [published Online First: 2010/05/11]

7. Kooiman J, van Hagen N, Iglesias Del Sol A, et al. The HAS-BLED Score Identifies Patients with Acute Venous Thromboembolism at High Risk of Major Bleeding Complications during the First Six Months of Anticoagulant Treatment. PLoS One 2015;10(4):e0122520. doi: 10.1371/journal.pone.0122520 [published Online First: 2015/04/24]

8. Nieto JA, Solano R, Ruiz-Ribo MD, et al. Fatal bleeding in patients receiving anticoagulant therapy for venous thromboembolism: findings from the RIETE registry. J Thromb Haemost 2010;8(6):1216-22.

9. Hach-Wunderle V, Bauersachs R, Gerlach HE, et al. Post-thrombotic syndrome 3 years after deep venous thrombosis in the Thrombosis and Pulmonary Embolism in Out-Patients (TULIPA) PLUS Registry. J Vasc Surg Venous Lymphat Disord 2013;1(1):5-12. doi: 10.1016/j.jvsv.2012.07.003 [published Online First: 2013/01/01]

10. van Dongen CJ, Prandoni P, Frulla M, et al. Relation between quality of anticoagulant treatment and the development of the postthrombotic syndrome. J Thromb Haemost 2005;3(5):939-42. doi: 10.1111/j.1538-7836.2005.01333.x [published Online First: 2005/05/05]

11. Ende-Verhaar YM, Cannegieter SC, Vonk Noordegraaf A, et al. Incidence of chronic thromboembolic pulmonary hypertension after acute pulmonary embolism: a contemporary view of the published literature. Eur Respir J 2017;49(2) doi: 10.1183/13993003.01792-2016 [published Online First: 2017/02/25]

12. Pengo V, Lensing AW, Prins MH, et al. Incidence of chronic thromboembolic pulmonary hypertension after pulmonary embolism. N Engl J Med 2004;350(22):2257-64. doi: 10.1056/NEJMoa032274 [published Online First: 2004/05/28]

13. Delcroix M, Lang I, Pepke-Zaba J, et al. Long-Term Outcome of Patients With Chronic Thromboembolic Pulmonary Hypertension: Results From an International Prospective Registry. Circulation 2016;133(9):859-71. doi: 10.1161/CIRCULATIONAHA.115.016522 [published Online First: 2016/01/31]

14. Office of National Statistics. National Life Tables, England 1980-82 to 2016-18 London, UK: Office for National Statistics; 2019 [Available from: https://www.ons.gov.uk/peoplepopulationandcommunity/birthsdeathsandmarriages/lifeexpectancies/bulletins/nationallifetablesunitedkingdom/2016to2018 accessed 01/04/2020.

15. Moore E, Munoz-Arroyo R, Schofield L, et al. Death within 1 year among emergency medical admissions to Scottish hospitals: incident cohort study. BMJ Open 2018;8(6):e021432. doi: 10.1136/bmjopen-2017-021432 [published Online First: 2018/07/02]

16. Fogelholm R, Murros K, Rissanen A, et al. Long term survival after primary intracerebral haemorrhage: a retrospective population based study. J Neurol Neurosurg Psychiatry 2005;76(11):1534-8. doi: 10.1136/jnnp.2004.055145 [published Online First: 2005/10/18]

17. Harenberg J, Roebruck P, Heene DL. Subcutaneous low-molecular-weight heparin versus standard heparin and the prevention of thromboembolism in medical inpatients. The Heparin Study in Internal Medicine Group. Haemostasis 1996;26(3):127-39. doi: 10.1159/000217198 [published Online First: 1996/05/01]

18. Hull RD, Schellong SM, Tapson VF, et al. Extended-duration venous thromboembolism prophylaxis in acutely ill medical patients with recently reduced mobility: a randomized trial. Ann Intern Med 2010;153(1):8-18. doi: 10.7326/0003-4819-153-1-201007060-00004 [published Online First: 2010/07/14]

19. Kleber FX, Witt C, Vogel G, et al. Randomized comparison of enoxaparin with unfractionated heparin for the prevention of venous thromboembolism in medical patients with heart failure or severe respiratory disease. Am Heart J 2003;145(4):614-21. doi: 10.1067/mhj.2003.189 [published Online First: 2003/04/08]

20. Ara R, Brazier JE. Using health state utility values from the general population to approximate baselines in decision analytic models when condition-specific data are not available. Value Health 2011;14(4):539-45. doi: 10.1016/j.jval.2010.10.029 [published Online First: 2011/06/15]

21. Pandor A, Horner D, Davis S, et al. Different strategies for pharmacological thromboprophylaxis for lower-limb immobilisation after injury: systematic review and economic evaluation. Health Technol Assess 2019;23(63):1-190. doi: 10.3310/hta23630

22. Chuang LH, Gumbs P, van Hout B, et al. Health-related quality of life and mortality in patients with pulmonary embolism: a prospective cohort study in seven European countries. Qual Life Res 2019;28(8):2111-24. doi: 10.1007/s11136-019-02175-z [published Online First: 2019/04/06]

23. Monreal M, Agnelli G, Chuang LH, et al. Deep Vein Thrombosis in Europe-Health-Related Quality of Life and Mortality. Clin Appl Thromb Hemost 2019;25:1076029619883946. doi: 10.1177/1076029619883946 [published Online First: 2019/12/17]

24. Enden T, Wik HS, Kvam AK, et al. Health-related quality of life after catheter-directed thrombolysis for deep vein thrombosis: secondary outcomes of the randomised, non-blinded, parallel-group CaVenT study. BMJ Open 2013;3(8):e002984. doi: 10.1136/bmjopen-2013-002984 [published Online First: 2013/08/31]

25. Luengo-Fernandez R, Gray AM, Bull L, et al. Quality of life after TIA and stroke: ten-year results of the Oxford Vascular Study. Neurology 2013;81(18):1588-95. doi: 10.1212/WNL.0b013e3182a9f45f [published Online First: 2013/10/11]

26. Marchetti M, Pistorio A, Barone M, et al. Low-molecular-weight heparin versus warfarin for secondary prophylaxis of venous thromboembolism: a cost-effectiveness analysis. Am J Med 2001;111(2):130-9. [published Online First: 2001/08/11]

27. Meads DM, McKenna SP, Doughty N, et al. The responsiveness and validity of the CAMPHOR Utility Index. Eur Respir J 2008;32(6):1513-9. doi: 10.1183/09031936.00069708 [published Online First: 2008/09/05]

28. Joint Formulary Committee. British National Formulary (online) [Drug Tariff]: London, UK; [Available from: http://www.medicinescomplete.com accessed 06/04/2020.

29. Menakaya CU, Pennington N, Muthukumar N, et al. The cost of outpatient venous thromboembolism prophylaxis following lower limb injuries. Bone Joint J 2013;95-B(5):673-7. doi: https://dx.doi.org/10.1302/0301-620X.95B5.30555

30. NHS Improvement. National Cost Collection: National Schedule of NHS Costs - Year 2018-19 - NHS trust and NHS foundation trusts London, UK2020 [Available from: https://www.england.nhs.uk/publication/2018-19-national-cost-collection-data-publication/ accessed 02/03/2020.

31. Curtis LAB, A.;. Unit costs of health and social care 2019 Canterbury, UK: Personal Social Services Research Unit; 2019 [Available from: https://www.pssru.ac.uk/project-pages/unit-costs/unit-costs-2019/ accessed 15/04/2020.

32. Luengo-Fernandez RY, G. S. C.; Gray, A. M.; Rothwell, P. M. Population-based study of acute- and long-term care costs after stroke in patients with AF. International Journal of Stroke 2012;8:308-14.

33. Curtis LAB, A.;. Unit costs of health and social care 2017 Canterbury, UK: Personal Social Services Research Unit; 2017 [Available from: https://www.pssru.ac.uk/project-pages/unit-costs/unit-costs-2017/ accessed 15/04/2020.

34. Grant PJ, Greene MT, Chopra V, et al. Assessing the Caprini Score for Risk Assessment of Venous Thromboembolism in Hospitalized Medical Patients. Am J Med 2016;129(5):528-35. doi: 10.1016/j.amjmed.2015.10.027 [published Online First: 2015/11/10]

35. Greene MT, Spyropoulos AC, Chopra V, et al. Validation of Risk Assessment Models of Venous Thromboembolism in Hospitalized Medical Patients. Am J Med 2016;129(9):1001 e9-01 e18. doi: 10.1016/j.amjmed.2016.03.031 [published Online First: 2016/04/25]

36. National Guideline Centre. Venous thromboembolism in over 16s - Reducing the risk of hospital-acquired deep vein thrombosis or pulmonary embolism: NICE guideline NG89. London, UK: National Institute for Health and Care Excellence, 2018.

37. Goodacre SH, K.; Shephard, N.; Pollard, D.; Hunt, B.; Fuller, G,; Nelson-Piercy, C.; Knight, M.; Thomas, S.; Lecky, F.; Cohen, J. Selecting pregnant or postpartum women with suspected pulmonary embolism for diagnostic imaging: the DiPEP diagnostic study with decision-analysis modelling. Heath Technology Assessment 2018;22(47)

38. Lubberts B, Paulino Pereira NR, Kabrhel C, et al. What is the effect of venous thromboembolism and related complications on patient reported health-related quality of life? A meta-analysis. Thromb Haemost 2016;116(3):417-31. doi: 10.1160/TH16-02-0152 [published Online First: 2016/07/01]
